# Graphene-based composites for heavy metal adsorption: a review on synthesis, mechanisms, and influencing factors

**DOI:** 10.1007/s11356-026-37947-x

**Published:** 2026-07-01

**Authors:** Avik Kumar Dhar, Piyas Halder, Nashita Ahmed, Ivan Moldavchuk, Maitry Bhattacharjee

**Affiliations:** 1 Department of Textiles, Merchandising, and Interiors, University of Georgia, 321 Dawson Hall, 305 Sanford Drive, Athens, GA 30602 USA; 2 Department of Textile Engineering, Shyamoli Textile Engineering College, Dhaka, 1207 Bangladesh; 3 Department of Environmental Science & Engineering, Bangladesh University of Textiles, Tejgaon, Dhaka 1208 Bangladesh; 4 Department of Chemical, Materials, and Biomedical Engineering, University of Georgia, Athens, GA 30602 USA

**Keywords:** Heavy metals, Adsorption, Graphene-based composites, Mechanisms, Factors, Thermodynamics, Regeneration

## Abstract

Heavy metal contamination in aquatic ecosystems is a global concern due to its toxicity, persistence, and potential for bioaccumulation. Effective removal is therefore essential. Among other techniques, adsorption is the most reliable and prevalent method of water purification. Graphene-based adsorbents exhibit significant advantages, including high specific surface area, small particle size, excellent mechanical properties, abundant functional groups, and high adsorption efficiency, making them promising materials for heavy metal removal from aqueous solutions. This article reviews recent advances in graphene and its derivative composites for heavy metal adsorption. It discusses structural features, adsorption mechanisms, synthesis methods, and the historical development of these composites. Key factors influencing adsorption capacity—such as temperature, pH, adsorbent dosage, contact time, initial metal concentration, organic ligands, and coexisting ions—are also examined. Additionally, adsorption thermodynamics and regeneration behavior are evaluated, and future research directions for practical applications are outlined.

**Graphical Abstract:**

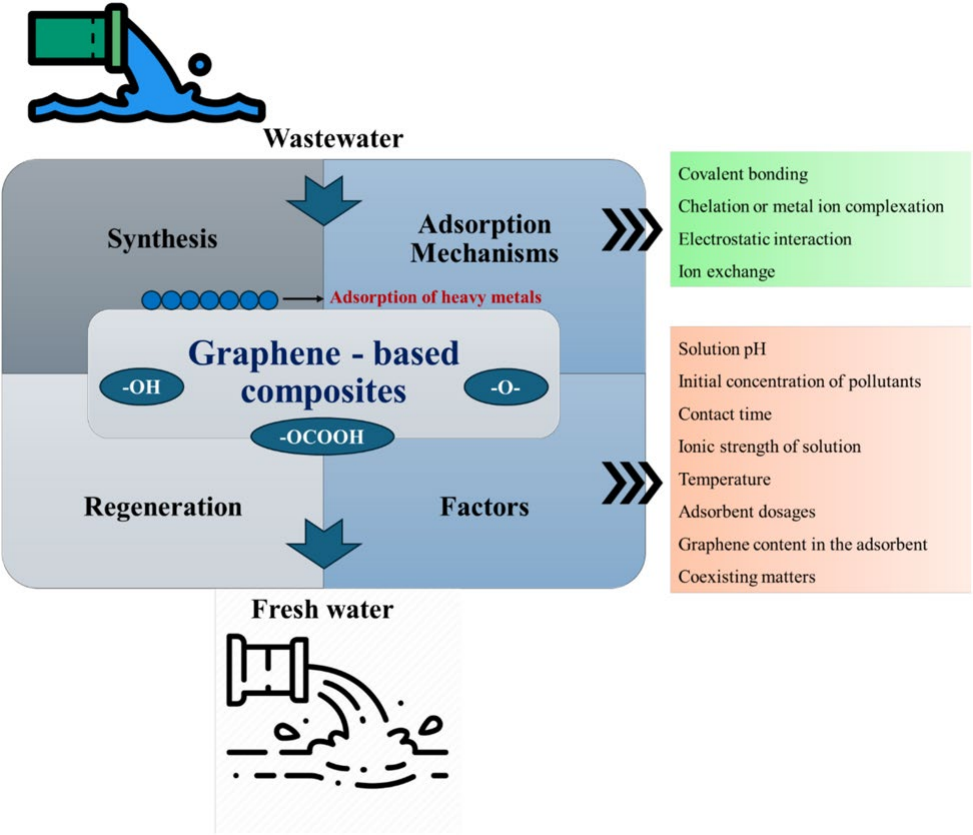

## Introduction

Life on Earth cannot exist without water. It is, however, seriously threatened by the massive pollution caused by domestic, agricultural, and industrial activities. Due to their bioaccumulation and poisonous nature, heavy metal contamination of water bodies poses serious consequences (Begum et al. 2021; Jagaba et al. 2024; Uthayakumar et al. 2022). Toxic heavy metal pollution poses a major threat to the environment because it cannot be biodegraded into non-hazardous or low-valence ions. As a result, these ions can accumulate in food chains. Therefore, it has emerged as a significant issue that has to be resolved immediately (Jiang et al. 2020; Karim et al. 2019). Numerous techniques have been documented so far for the removal of heavy metals from aqueous systems, including chemical precipitation (Benalia et al. 2022), membrane separation (Kasuri et al. 2026), electro-coagulation (Jain et al. 2022), and ion (cations and anions) exchange (Pan et al. 2021). Unfortunately, these technologies have several drawbacks that limit their practical applications (Maneechakr & Mongkollertlop 2020).

Adsorption is the most promising technique for heavy metal removal due to its low cost and high efficiency. Among different adsorbents, carbon-based adsorbents are particularly appealing because of their superior mechanical and chemical stability, large specific surface area, and variable surface functional groups (Nayeem et al. 2023; Lv et al. 2024). Diverse carbon-based adsorbents, including activated carbon, CNTs, and biochar, are frequently deployed commercially (Yang et al. 2019). The main drawbacks of utilizing activated carbon are its high regeneration costs and column fouling. Therefore, research into more potent alternative carbon-based adsorbents is still ongoing. CNTs and graphene, two nanocarbonaceous materials, have gained appeal as promising adsorbents in the past decade. In contrast to other materials, graphene and its derivatives have sparked a revolution in heavy metal adsorption due to their exceptional characteristics, including a high surface area, rapid adsorption kinetics, remarkable physicochemical stability, superior mechanical strength, and outstanding thermal and electrical conductivity (Table 1). Additionally, graphene’s production costs are significantly cheaper than those of CNTs and other adsorbents, which make them an ideal candidate for heavy metal adsorption (Mbayachi et al. 2021; Melchor-Durán et al. 2024; Rahaman et al. 2024; Thi Mai et al. 2024).

**Table 1:** Summary comparison table on Pb (II) and Cd (II) adsorption

Pollutants	Graphene-based adsorbents	Activated carbon (AC)	Biochar (BC)
Pb (II)	117.6–344.8 mg/g (GO/rGO) and up to 2503.6 mg/g (rGO hydrogel) (Kaptanoğlu and Yuşan 2022; Asemaneh et al. 2020; Hu et al. 2022)	81.9–130 mg/g (plant-based AC) (Al-Ma’abreh et al. 2024; Kongsune et al. 2021)	125.2 mg/g (GO-supported BC), up to ~ 1641 mg/g in CNT-BC hybrids) (Billah et al. 2025; Yang et al. 2020)
Cd (II)	312.5 mg/g (GO-PAN composite), 1185.3 mg/g (rGO hydrogel) (Asemaneh et al. 2020; Hu et al. 2022)	117.3 mg/g (AC cloth) (Al-Ma’abreh et al. 2024)	98–327 mg/g (modified BC), lower for pristine BC (Xu et al. 2024; Qi et al. 2022)

For instance, fungal hyphae-derived nanobiocomposites incorporating GO demonstrated outstanding removal efficiencies, surpassing 90% for both Cr (VI) and Ni (II) (Yadav et al. 2024). Furthermore, in another study, the incorporation of GO enhanced the adsorption capacity of GO-CMC-PVA composite membranes for aqueous heavy metals, achieving maximum values of 262.1, 237.9, 319.3, and 413.6 mg/g for Ni (II), Cu (II), Ag (I), and Pb (II), respectively (Zheng et al. 2024). In addition, a sulfonated and triethylenetetramine-functionalized GO-chitosan adsorbent (T-SGO-CS) was synthesized and applied for heavy metal removal from single, binary, and ternary systems, exhibiting adsorption capacities of 312.3, 260.5, and 84.6 mg/g for Pb (II), Cd (II), and Ni (II), respectively (Peng et al. 2024). Additionally, the PG-GO-PEI composite showed nearly 100% removal efficiency, with a maximum Cr (VI) adsorption capacity of 313.5 mg/g (Hu et al. 2025). Although extensive research exists in this field, the lack of a concise, integrated review motivates this literature review. This review compared recent advances in heavy metal adsorption by graphene and its derivative composites. The initial part of this review presented a focused analysis of composite structures, synthesis methods, and adsorption mechanisms. Key adsorption parameters—including temperature, pH, dosages, contact time, metal concentration, organic ligands, and competing ions—were critically analyzed. Furthermore, adsorption thermodynamics, regeneration behavior, and prospects on practical applications of graphene-based adsorbents were also highlighted. Table 2 summarizes the recently published review papers closely aligned in this area.

**Table 2:** Recently published review articles closely related to this field

Adsorbent types	Topic covered	Target pollutants	Ref
GO-based composites	Synthesis route, mechanism, isotherm, and kinetic model	Heavy metal	(Nirmala et al. 2022)
Graphene composites	Isotherm, kinetics, thermodynamics, and applications	Heavy metal	(Parvathi et al. 2023a, b)
CNT and graphene-based nanomaterials	Fundamental concepts, mechanisms, functionalization, MD simulation, and DFT	Heavy metal	(Chandran et al. 2023)
Graphene-based materials	Functionalization, morphology, and structure	Heavy metal	(Kong et al. 2021)
Graphene-based materials	Preparation, modification, and factors	Heavy metal	(Xu et al. 2022)
Graphene-based nanomaterials	Toxicity, roles of functional groups, and mechanism	Heavy metal	(Ahmad et al. 2020)
Functionalized graphene-based composites	Synthesis, properties, factors, and mechanisms	Heavy metal and organic pollutant (dyes)	(Iqbal et al. 2023)
GO-based adsorbent	Production, mechanisms, kinetics, isotherms, and thermodynamics	Heavy metal and dye in textile processing	(Velusamy et al. 2021)
Graphene and its derivative composites	Graphene and derivatives, composites, properties, adsorption mechanisms, factors, thermodynamics, regeneration, and outlook	Heavy metal	This work

## Graphene and its derivatives

**Fig. 1:**
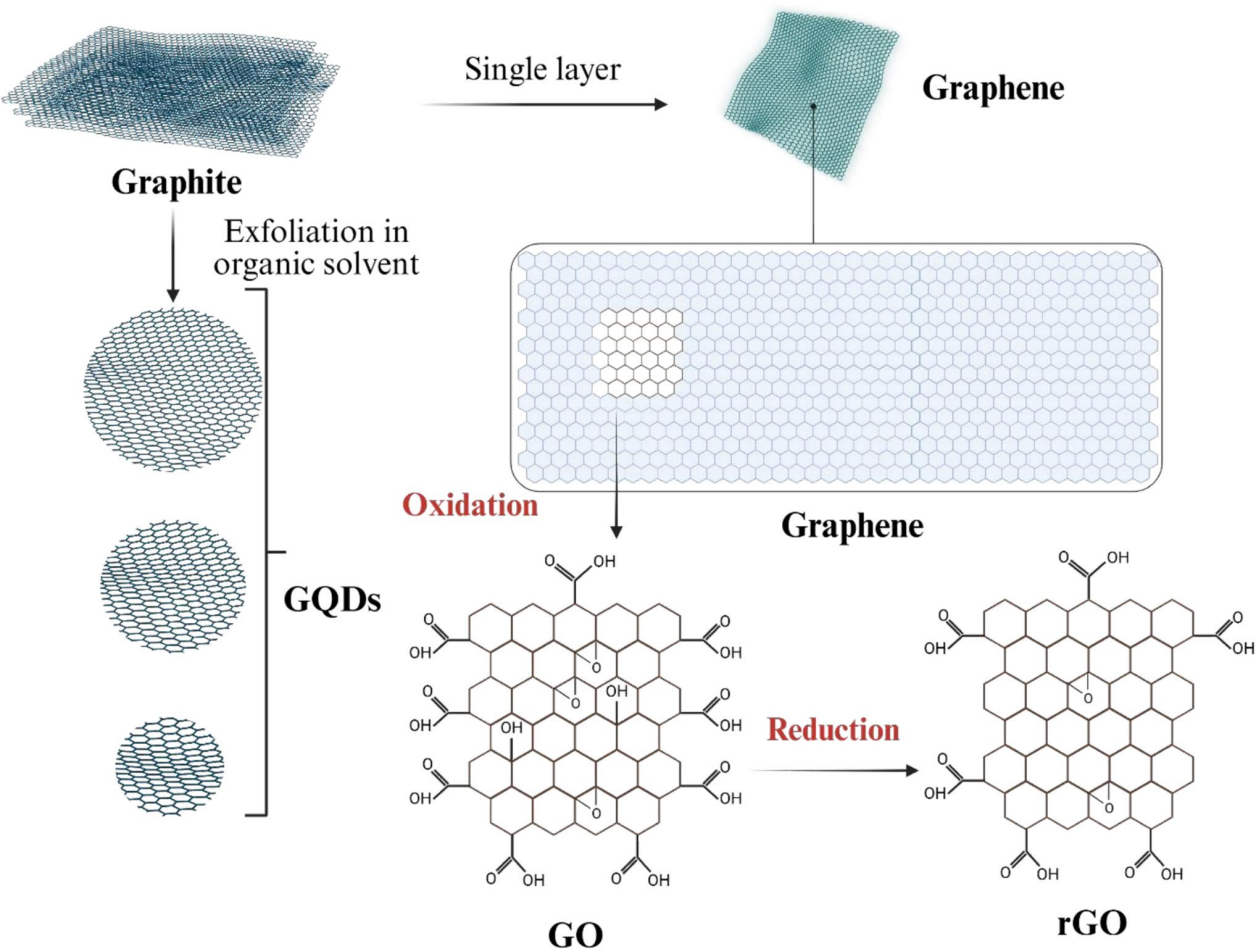
Graphene and its derivatives for heavy metal adsorption

Graphite is the most widely used allotropic of carbon. In graphite, van der Waals forces are employed to stack layers of sp^2^-hybridized carbon elements. In contrast, graphene is a single sheet of carbon atoms firmly packed into a 2D honeycomb crystal structure (Ge et al. 2019; Li et al. 2019). Supercapacitors, optical materials, biological applications, electrically conductive materials, and other fields have made extensive use of graphene due to its distinctive features, including its large specific surface area and electrical conductivity (Geim & Novoselov 2007). Because of its in-plane nature, it is highly conductive in a direction parallel to graphene layers, but, because of the weak van der Waals forces interacting among them, it exhibits poor conductivity in a direction perpendicular to the layers (Almoisheer et al. 2019; Cao et al. 2021; Thakur & Kandasubramanian 2019). Figure 1 demonstrates the common graphene derivatives used in heavy metal adsorption.

Functionalization of graphene and its derivatives is crucial for heavy metal adsorption since it induces specific functional groups (such as thiol, amine, and carboxyl), which increase the number and diversity of binding sites for heavy metals (Alimohammady et al. 2025). For example, thiol-functionalized GO demonstrated adsorption capacities of 116.9 mg/g for Pb (II) at pH 6 and 112.9 mg/g for Cd (II) at pH 7. The high adsorption capacity was attributed to the abundant oxygen and sulfur functional groups present on functionalized GO (Bhardwaj et al. 2024). In another study, sulfur nanoparticles (SNPs) were uniformly loaded onto the surface of GO to fabricate GO-S. Enhanced by the thiophilic nature of heavy metal ions, GO-S exhibited adsorption capacities 1.7–12.4 times higher and achieved 94–98% removal of Cu (II), Cd (II), Hg (II), Pb (II), and Zn (II) within 1 h (Fan et al. 2025). The following sections will introduce the history and structure of graphene and graphene derivatives for heavy metal adsorption in aqueous systems.

### Pristine graphene

Since Geim and Novoselov discovered graphene, the material has garnered significant attention globally for its potential applications in various sectors, including electrical, transportation, electrochemical, medical, supercapacitor, energy, photochemical, and optical devices (Bhagavathsingh et al. 2023; Novoselov et al. 2004; Yan et al. 2024). However, the quality and performance of graphene are significantly influenced by synthesis methods. Table 3 summarizes the current approaches for synthesizing graphene.

**Table 3:** Current approaches to synthesizing graphene

Synthesis techniques	Conditions	Advantages	Disadvantages	Ref
Mechanical exfoliation	Normal and lateral forces, and solvent selection	High-quality graphene, scalability	Low monolayer yield and fragmentation effect	(Liu et al. 2021; Moosa & Abed 2021)
Liquid phase exfoliation	Solvent selection, sonication	Scalability, flexibility in terms of deposition on the substrate	Solvent limitations and surface energy matching	(Moosa & Abed 2021)
Electrochemical exfoliation	Electrolyte selection, electric force	High-quality graphene, scalability	Complexity of reaction mechanism, solvent stability	(Moosa & Abed 2021)
CVD	Precursor and substrate. Temperature and pressure	Can produce large and high-quality graphene films	Substrate interaction and uniformity	(Abbas et al. 2022; Saeed et al. 2020)
Roll-to-roll synthesis	Continuous process, uses CVD	Long film production, scalability	Complex process, needs equipment investment	(Bubnova 2021)
Chemical reduction	Reducing agents, temperature, pH	Cost-effective, scalable, simple	Toxic reducing agents, environmental impact	(Deshmukh et al. 2026)
Epitaxial growth	Need a substrate for reaction and high-temperature annealing	Can produce large and high-quality graphene films	Limited substrate availability and complexity of the process	(Sun et al. 2022)
Laser ablation	Laser parameters, ablation medium	High-quality graphene, simplicity, and a green process	Controlling nanostructure size might be difficult, as medium-dependent properties	(Altuwirqi 2022)

### Graphene oxide (GO)

**Fig. 2:**
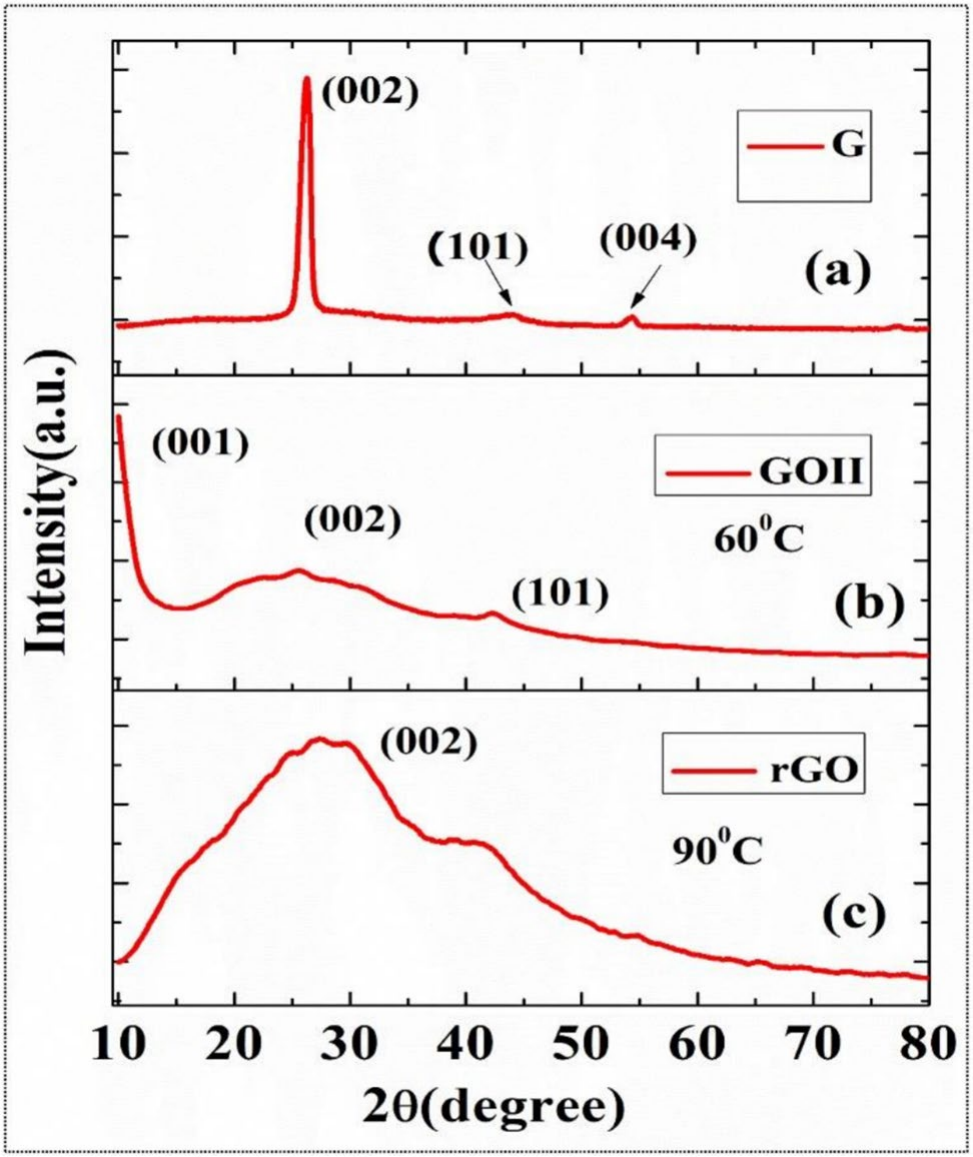
XRD curve of (**a**) graphene, (**b**) GO, and (**c**) rGO. Reproduced with permission from Patil et al. (2026)

GO represents graphene in its oxygenated form. Benjamin Brodie synthesized GO in 1859, a long time before pure graphene was discovered. Referring to it as a novel carbon-based substance, he named it carbonic acid. Between 1859 and 1958, several synthesis techniques were claimed. These techniques mostly consisted of the Brodie, Staudenmaier, and Hummers processes, which are the main paths for synthesizing GO (Brodie 1859; Geim 2011, 2012; Hummers & Offeman 1958; Staudenmaier 1898). Among them, Hummers’ approach has drawn the most attention due to its widespread use and familiarity in adsorption. Later, Hummers’ approach underwent major modifications (the Tour, Peng, and 4-step methods) to improve functionality and reduce harmful gas emissions (Pendolino et al. 2015; Peng et al. 2015; Sun & Fugetsu 2013). Recently, a modified Hummers method was used to synthesize GO and rGO from graphene nanoplatelets rather than graphite. XRD analysis revealed a sharp (002) peak for graphene at 26.34°, a strong (001) peak for GO at 10.05°, and a broad (002) hump for rGO at 25.5°, confirming oxidation and partial recovery of the graphite structure (Fig. 2) (Patil et al. 2026).

Furthermore, GO is used as the starting material for synthesizing other derivatives, including fluorographene, bromographene, graphane, and many others, due to the presence of various oxygen-containing functional groups on its surface (Jiříčková et al. 2022). Its exceptional features include a 2D single atomic layer structure, a multitude of functional groups (-OH, -COOH, -C = O, and others), strong affinity for metal ion adsorption, chemical stability, fast adsorption rate, and lower production costs (Neo et al. 2020; Park et al. 2024; Sahu et al. 2022). In addition, the π-π electrostatic interaction of GO offers a plethora of binding sites. To develop a graphene composite with multiple surface chemistries and improved metal binding performance, additional sulfur and amino functional groups can be added through the covalent interaction of cysteamine on the sp^2^ carbon of GO via the thermal thiol-ene click reaction (Yap et al. 2019).

### Reduced graphene oxide (rGO)

Another significant derivative of the graphene family is reduced graphene oxide (rGO). Because of its reduced form, it is less negatively charged than GO because it has fewer remaining oxygenated functional groups (Hossain et al. 2020). The process of developing rGO typically involves treating GO with reducing agents, thermal treatment, UV light, and electrochemical reduction to eliminate various functional groups from GO (Budiarso et al. 2024; Doğan et al. 2026; Adhikary and Goswami 2025; Agarwal and Zetterlund 2021).

### Graphene quantum dots (GQDs)

GQDs represent an additional derivative of graphene that can provide further functionality for the adsorption of heavy metals because of their edge morphology and quantum confinement (Haque et al. 2023; Nuengmatcha 2021; Wang et al. 2022). GQDs have been developed through a variety of techniques, which include the following:

- Top-down: breaking down bulk materials into nanostructured materials (example: chemical exfoliation, nanolithography, electrochemical cutting, and solvothermal preparation).
- Bottom-up: formation of bigger units from small units (example: hydrothermal heating, thermal combustion, microwave irradiation, and cage-opening technique) (Biswas et al. 2021).

**Fig. 3:**
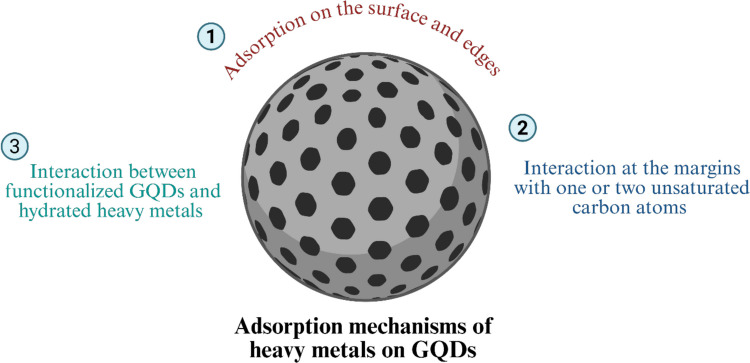
Adsorption mechanisms of heavy metals on GQDs

The following (Fig. 3) are the mechanisms by which the hydrated heavy metals are adsorbed on GQDs (Abdelsalam et al. 2019).

### Recent advances in synthesis techniques

Recent developments in graphene and graphene-derivative synthesis strongly emphasize cost-effectiveness, sustainability, and scalability, with several approaches showing clear industrial promise. Green reduction strategies have advanced rapidly, with ascorbic acid, plant extracts such as shallots, and agricultural-waste-derived reagents enabling low-cost, environmentally benign production of rGO while maintaining good structural quality (Parvathi et al. 2023a, b; Sarkar et al. 2026). Simplified one-pot and hydrothermal reduction pathways further enhance throughput and safety by minimizing steps and avoiding hazardous chemicals (Wang et al. 2021a, b; Ramesh et al. 2023). High-throughput methods such as microwave-assisted thermal reduction also repair defects efficiently, improving conductivity and facilitating large-scale manufacturing (Park et al. 2022). Beyond chemical routes, emerging reactor- and plasma-based methods enable continuous, catalyst-free graphene generation at reduced energy inputs, while flash joule heating offers a transformative high-yield route capable of converting inexpensive carbon sources directly into graphene (Wyss et al. 2021). Biomass and waste-derived feedstocks, including coal, biochar, battery waste, and agricultural residues, provide additional low-cost pathways, aligning sustainability with scalable production goals (Tamuly et al. 2022; Freitas et al. 2020). Recent demonstrations of industrial-scale synthesis, such as thermal plasma spraying, achieving high-quality rGO at 52 g/h and 4.36 USD/g, highlight the practical viability of these approaches for future commercialization (Kumar et al. 2025).

## Graphene-based composites for heavy metal adsorption

**Fig. 4:**
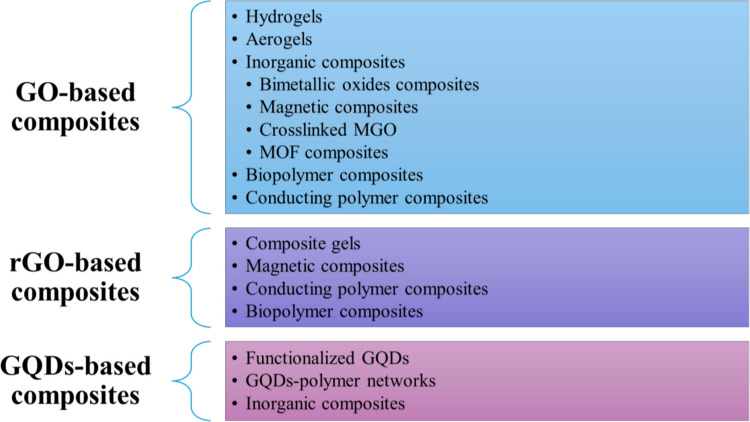
Graphene and its derivative-based composites for heavy metal adsorption

**Fig. 5:**
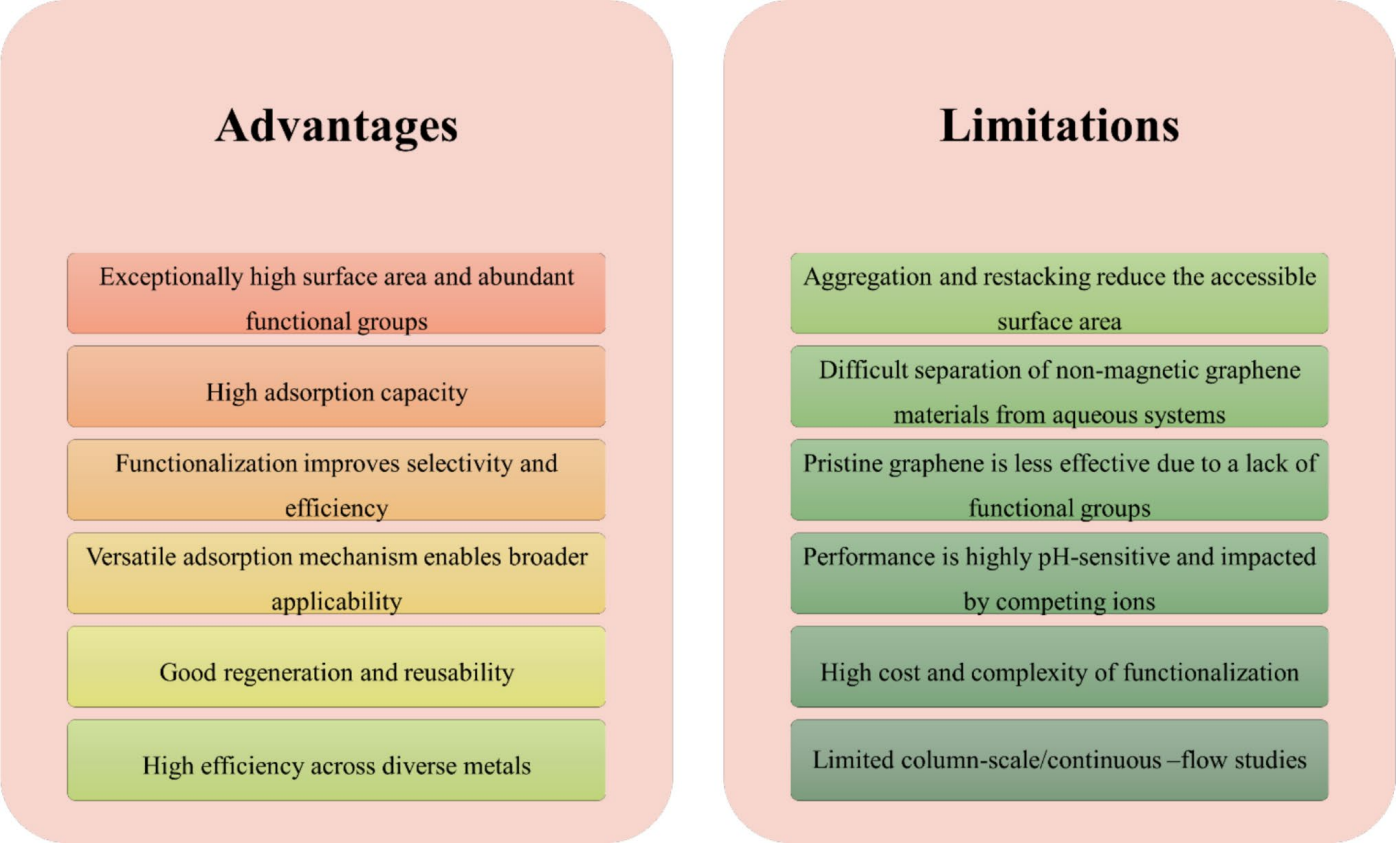
Advantages and limitations of graphene-based composites for heavy metal adsorption

Due to its simple carbon structure and the absence of other functional groups, pure graphene exhibits very low water uptake, resulting in a moderate adsorption capacity and reduced selectivity under realistic aqueous conditions. In contrast, GO appeared to be the most worthy of further investigation among the different carbonaceous compounds. The oxygen-containing surface functional groups (− OH, − COOH, etc.) of GO are key factors (Lestari et al. 2025; Mokoena & Mofokeng 2023). This section will cover the recent developments in the synthesis of graphene and its derivative composite adsorbents for heavy metal adsorption. Figure 4 summarizes the graphene and its derivatives-based composite materials for heavy metal adsorption, and Fig. 5 demonstrates the advantages and limitations of graphene-based composites.

### GO-based composites

#### Hydrogels

**Fig. 6:**
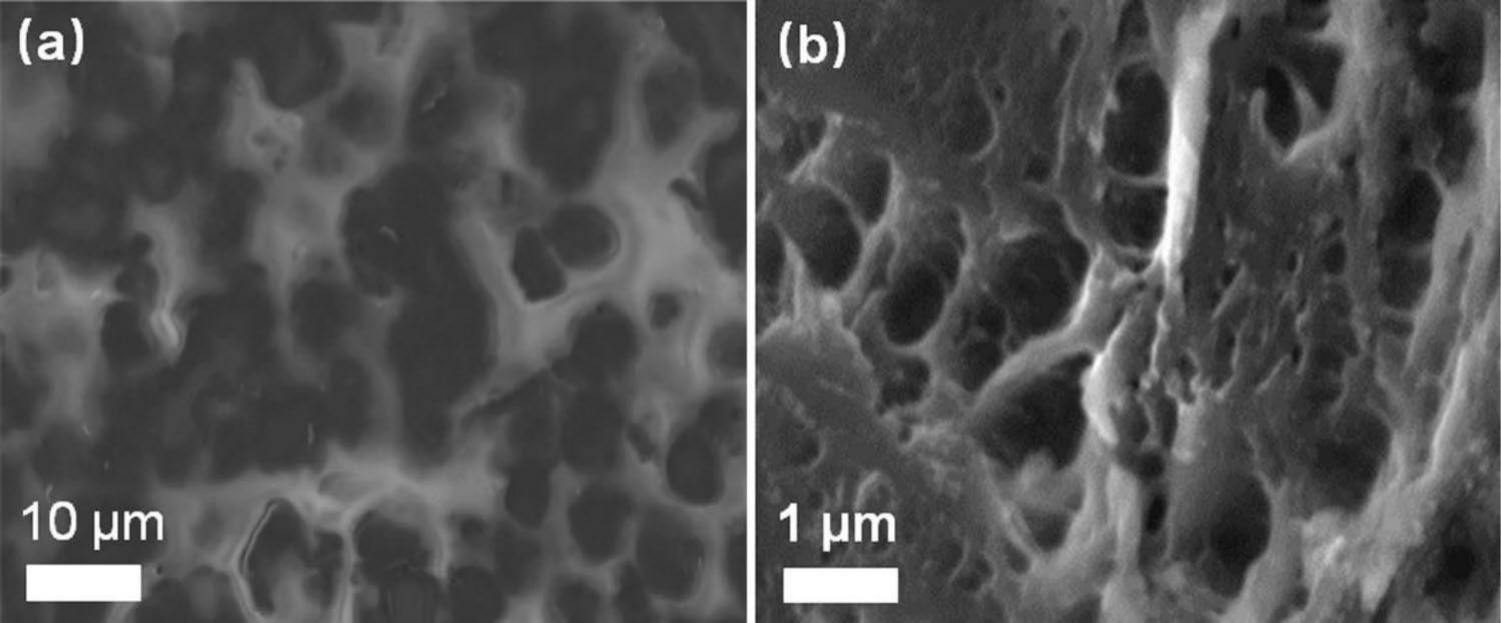
SEM images of the porous GO-g-P4VP@PAA hydrogel. Reproduced with permission from Zhang et al. (2023)

GO-based composite hydrogels are synthesized by incorporating GO into various polymeric matrices to enhance the adsorption properties for heavy metal removal. These hydrogels can be prepared via physical or chemical crosslinking (Feng et al. 2022; Sharma et al. 2025). Furthermore, incorporation of a tiny amount of GO into the hydrated polymer network enhances the mechanical properties of the composite hydrogels. However, the broad conjugated planar structure and the finite oxygen-containing surface functional groups greatly restrict the surface activity of GO and limit its compatibility with the matrix of many polymers. Therefore, polymer brushes are grafted on the GO surface to improve functionality. For example, the incorporation of PAA and P4VP brush improved compressive strength and imparted an interconnected 3D mesoporous structure in GO-g-P4VP-PAA composite hydrogel (Fig. 6a and b shows the cross-sectional images of the composite hydrogel at different magnifications). Furthermore, the composite hydrogel achieved maximum adsorption capacities of 257.3 mg/g for Pb (II) and 175.8 mg/g for Cd (II) (Zhang et al. 2023).

The GO layers in conventional GO-biopolymer composites are physically entrapped within the structure and are less stable. However, the stability and adsorption capacity of GO-based adsorbents can be enhanced through the functionalization of GO. For instance, crosslinking the GO layers using multifunctional PEI improved stability, efficiency, and reusability of the hybrid structure. Thus, functionalized GO embedded in a calcium alginate matrix showed significantly better removal of Pb (II), Hg (II), and Cd (II) ions (maximum uptake of 602.0, 374.0, and 181.0 mg/g, respectively) from aqueous solution (Arshad et al. 2019).

#### Aerogels

**Fig. 7:**
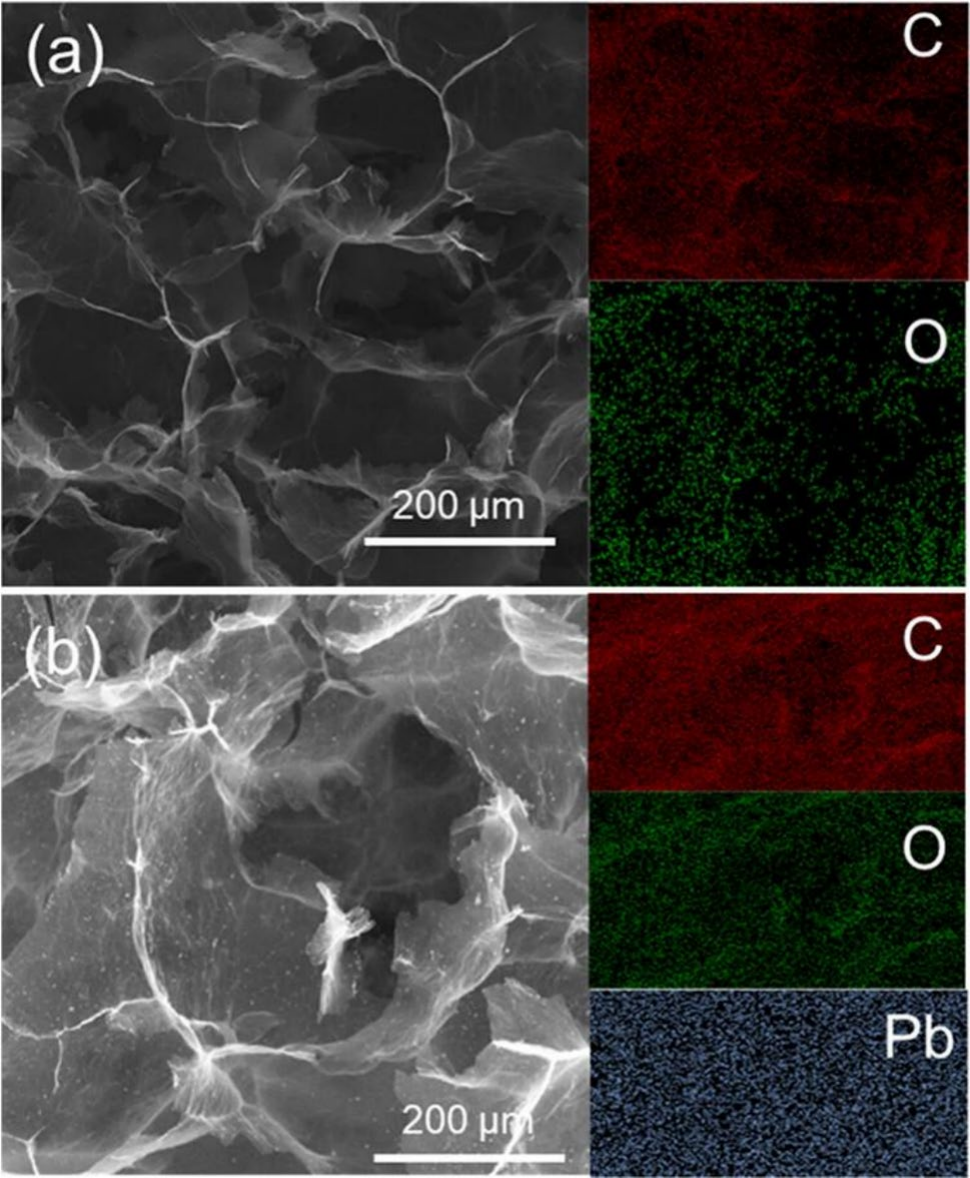
SEM and EDS images of DPTA-functionalized GO/CMC aerogel (**a**) before and (**b**) after adsorption. Highly intricate pore network of aerogel (pore size range: 70–130 μm). Reproduced with permission from Gou et al. (2024)

Incorporating graphene and CNTs into hybrid aerogels, novel porous materials with specific adsorption properties and desired structures were developed. Hydrothermal reduction was reported as a green, viable approach to fabricating ultra-lightweight 3D graphene-MWCNT-PDA hybrid aerogels. Incorporation of MWCNT-PDA allowed structural stability, efficiently inhibited the stacking of GO sheets, and exposed additional active sites to enhance adsorption capacity (for Cu (II) 318.5 and Pb (II) 350.9 mg/g) (Zhan et al. 2019). Gou et al. (2024) developed a DPTA-functionalized GO/CMC aerogel with abundant chelating groups for highly selective Pb (II) adsorption. It achieved a notably high capacity of 521.9 mg/g and strong selectivity driven by DPTA-Pb (II) complexation, GO’s oxygen-rich functional groups, and a highly intricate pore network (Fig. 7) (Gou et al. 2024).

GO-SF hybrid aerogels were synthesized via a freeze-drying technique, with SF acting as a cross-linker to aid in the assembly of the GO-based network. The pores in the aerogels played a significant role in the diffusion of hazardous compounds and had a high Ag (I) adsorption capacity. The incorporation of SF changed the labile functional groups into stable groups, making the hybrid hydrogel more thermally stable. Hydrogen bonding between oxygen functional groups of GO and amide groups of SF, as well as hydrophobic interactions between GO and crystalline β-sheets, improved the thermal stability of the GO-SF aerogels (Wang et al. 2019). Table 4 summarizes the recent research on GO-based composite gels for heavy metal adsorption.

**Table 4:** Properties of GO-based composite gels for heavy metal adsorption

GO-based composite gels	Target heavy metals	Adsorption capacity	Key functional groups/modifications	Adsorption mechanisms	Notable features	Ref
Amine-modified GO hydrogel (UCN-GH)	Cu (II)	2.0–2.5 mmol/g	Amine groups (-NH_2_, -NH-), GO	- Coordination - H-bonding - Electrostatic	- Enhanced adsorption by amine modification	(Feng et al. 2022)
Thiol and amido modified alginate-based GO hydrogel	Pb (II) Cu (II)	369.6 mg/g 124.1 mg/g	From modified alginate: -OH, -COOH, -SH, -NH_2_ From GO: -OH, -COOH	- Coordination - Chelation - Ion exchange - Surface complexation - Electrostatic interaction	- Novel combination - Structural stability - Rugged surface - Optimal size: 2.5 mm wet, 0.5 mm dry	(Zhang et al. 2022)
Calcium alginate—GO hydrogel beads	Cr (III) Cu (II) Cd (II)	90.6 mg/g 108.6 mg/g 134.8 mg/g	Alginate crosslinked with Ca^2+^; GO providing COOH, OH, epoxy groups	- Electrostatic interaction - H-bonding - Dipole–dipole interactions - van der Waals forces - Ion exchange	- Ecofriendly - Stable 3D porous structure - Reduce GO aggregation	(Ahmed et al. 2024)
GO—montmorillonite composite aerogel (slit-pore structure)	Cu (II)	95.1% removal efficiency	GO with abundant oxygenated groups; MMT layered structure; crosslinking increases pore density	- Electrostatic interaction - Coordination via oxygen functional groups, enhanced by slit-shaped pores	- Aerogel overcomes GO aggregation - High porosity - Improved Cu selectivity	(Hao et al. 2022)
GO–Gd_2_O_3_-biopolymer gel granules (SA-CMC matrix)	Cr (III) Pb (II) As (V)	29.2 mg/g 158.7 mg/g 36.8 mg/g	GO with COOH/OH/epoxy groups plus Gd_2_O_3_ (strong affinity to oxyanions); biopolymer gel (alginate + CMC)	- Surface complexation - Electrostatic interaction - Metal–ligand coordination	- Stable granules - Removes both anionic and cationic metals - Reusable - Suitable for real water	(Lee et al. 2022)
GO-PANI composite	Pb (II)	1416.0 mg/g	Aniline grafted, then PANI polymerized on GO edges; amine-rich groups	- Chelation/coordination with amines - Electrostatic attraction	- Extremely high Pb (II) capacity - PANI-GO synergy	(Xie 2023)
Carboxylated GO-chitosan composite hydrogel	U (VI) Pb (II) Cr (VI) Cd (II)	64.9 mg/g 384.6 mg/g 68.5 mg/g 49.5 mg/g	Carboxylated GO; chitosan provides -NH_2_ sites	- Complexation with amine and oxygen groups	- Microwave synthesis - High removal (90–95%) - Reusable	(Patel et al. 2022)

#### Inorganic composites

Integrating inorganic nanoparticles with GO improves the physical and chemical properties of the composites. For example, GO-SiO_2_ nanocomposite revealed a remarkable ability to kill pathogens and demonstrated a maximum adsorption capacity of 181.8 mg/g for Cr (VI). The inclusion of SiO_2_ nanoparticles between the GO layer structure altered the internal structure of GO, resulting in a more porous structure. Besides, nanoparticles formed a hollow gap between the GO layers to improve the adsorption capacity (Dan et al. 2023). The following section discusses the recent progress in bimetallic oxides, magnetic composites, and crosslinked MGO composite for heavy metal adsorptions.

##### Bimetallic oxide composites

**Fig. 8:**
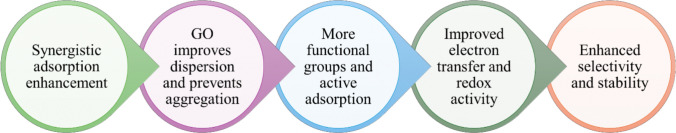
Key advantages of GO-based bimetallic composites for heavy metal adsorption

Metal oxides are commonly used for heavy metal adsorption because of their good chemical stability, non-toxicity, and low cost. The weak regeneration and low uranium adsorption capability of single metal oxides limit their use. Bimetallic oxides not only retain the qualities of single-metal oxides but also offer distinct benefits, including high surface activity and chemical stability, thereby broadening their practical applications. Fig. 8 demonstrates the advantages of GO-based bimetallic composites for heavy metal adsorption. However, bimetallic oxides are easily aggregated in solutions, resulting in reduced adsorption efficacy (Zhang et al. 2019a, b).

To overcome metal oxide agglomeration, bimetallic oxides containing chelating groups are crosslinked with GO. In recent work, GO-supported Ti_x_Al_1-x_O_y_ bimetallic oxide was synthesized utilizing the sol–gel technique. Ethylene glycol was used to complete hydrolysis and liberate crosslinking. Incorporation of Ti_x_Al_1-x_O_y_ formed a porous network, and the nanoparticles were evenly disseminated on the surface of GO. In static adsorption studies, the adsorption efficiency and capacity of GO-Ti_x_Al_1-x_O_y_ achieved 97.8% and 614.6 mg/g at 25 °C and pH 4 (Ding et al. 2023). Table 5 summarizes the GO-based bimetallic oxide systems used for heavy metal adsorption.

**Table 5:** Bimetallic oxide-based systems and their functions for heavy metal adsorption

System	Components	Applications	Ref
Sn_4_CoO_9.3_-GO	Bimetallic Sn-Co oxide + E rGO	Pb (II) and Hg (II) detection and enrichment	(Zeng et al. 2024)
LaFeO_3_-GO	Perovskite La-Fe oxide + GO	PO_4_^3−^ adsorption, model for bimetal-GO synergy	(Rauf et al. 2024)
Mg–Al LDH-GO	Layered double hydroxide (bimetal) + GO	Dye and heavy metal removal	(Mahar et al. 2026)
CulnS_2_-Ni-MoS_2_-NrGO	Mixed metal sulfides + rGO	Cr (VI) reduction and adsorption	(Mishra et al. 2026)
Pb–Zn-MOF-GO	Bimetallic MOF integrated with GO	Pb (II) and Zn (II) adsorption	(Liu et al. 2024)

##### Magnetic composites

**Fig. 9:**
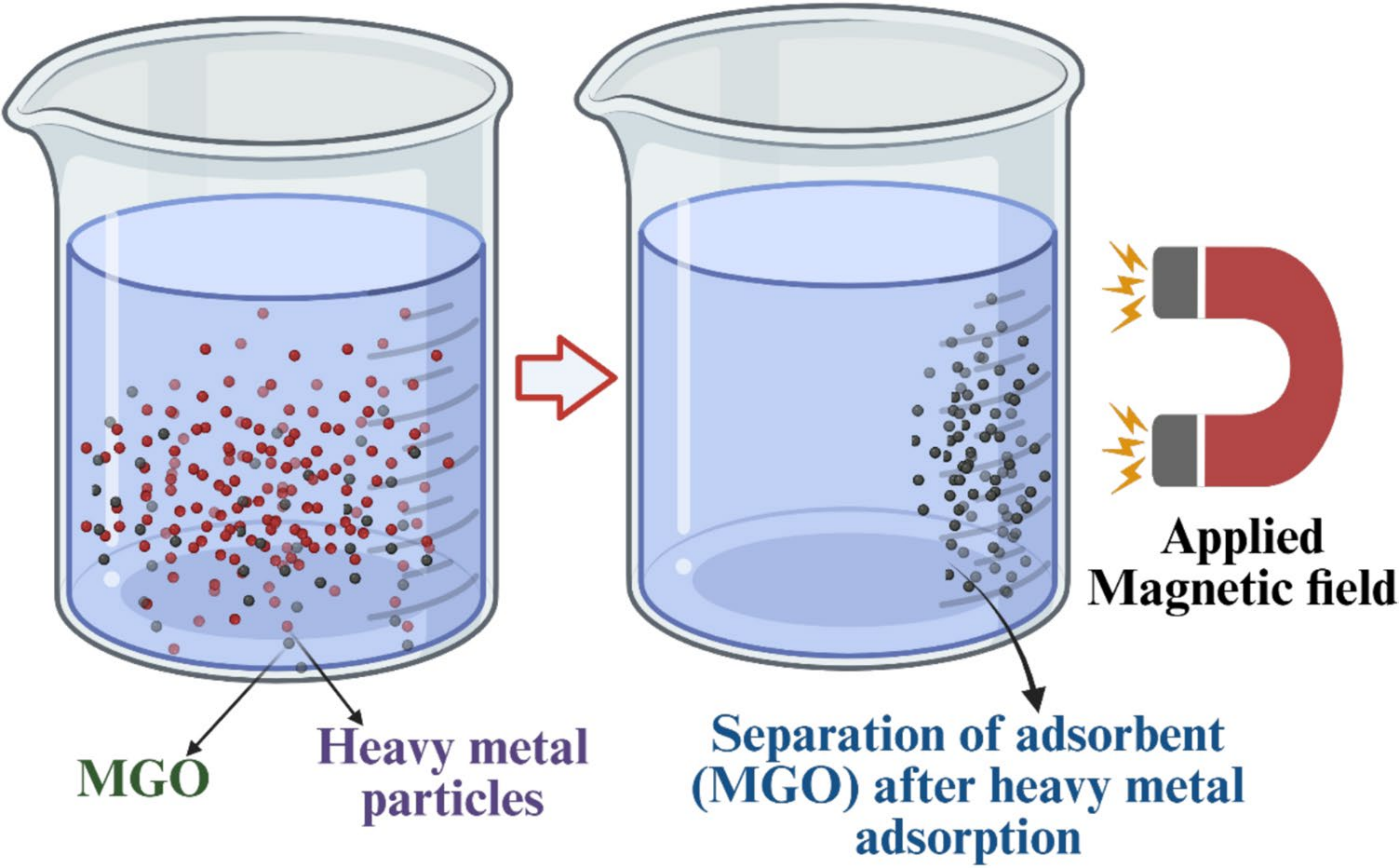
Separation of MGO after heavy metal adsorption

Pure GO has difficulties in separating from the solution and requires centrifugation and filtration for after-use separation. The use of GO-based magnetic adsorbents is a solution in this perspective (Fig. 9). Magnetic composites integrate Fe_3_O_4_ or related ferrites with GO to add redox-active Fe–O sites, faster electron/ion transfer, improved dispersion, and prevention of GO restacking; these features enhance chemisorption and multi-site binding along with magnetic separability (Bulin et al. 2020; Wu et al. 2019; Li et al. 2022a, b). GO was coated with iron particles in the co-precipitation method, and the developed MGO demonstrates spontaneous and endothermic adsorption of Pb (II), Cr (II), Cu (II), Zn (II), and Ni (II) with the maximum adsorption capacities of 200.0, 24.3, 62.9, 63.7, and 51.0 mg/g, respectively. Furthermore, it exhibited good antibacterial properties, with maximal removal efficiencies of 98.79%, 97.15%, and 97.69% for *E. coli*, *Y. ruckeri*, and *Antobacter agglomerans*, respectively (Farooq & Jalees 2020). A further investigation utilized a modified MGO-ionic liquid to adsorb As (III) and As (V) from an aqueous mixture, with maximal adsorption capacities of 160.7 mg/g for As (III) and 104.1 mg/g for As (V) (M. Zhang et al. 2019a, b).

The synthesis of MGO generally relies on synthetic graphite powder as the starting material, while the use of natural graphite sources for GO synthesis has been relatively little explored (Tewatia et al. 2021). Recently, Cr (VI) adsorption was investigated utilizing a GO-Fe_3_O_4_ composite based on natural graphite derived from kusambi wood (Timor Island, Indonesia). GO was synthesized using Hummer’s modification approach, whereas GO-Fe_3_O_4_ was synthesized through the in situ co-precipitation method. The optimum adsorption capacity was investigated at the usage of 0.1 g of adsorbent under acidic conditions (pH 2) for 80 min at 25 °C (Neo et al. 2020). Table 6 summarizes the recent research on the properties and performance of GO-based magnetic composites.

**Table 6:** Properties and performance of GO-based magnetic composites

Composites	Target metals	Key properties	Optimal adsorption conditions	Adsorption performance	Ref
rGO + magnetic nanoparticles	Hg (II)	- Magnetic separation - rGO support	pH—6 Time—100 s Temp—25 °C Adsorbent—0.05 g	9.15 μg/L Hg residual under optimal conditions	(Babaei et al. 2025)
GO + Fe_3_O_4_	Cr (VI)	- Large surface area - Magnetic	Adsorbent—0.1 g pH—2 Time—80 min Temp—25 °C	3.197 mg/g	(Neolaka et al. 2020)
Chitosan – Fe_3_O_4_-GO	Ni (II)	- Chitosan increases binding sites - Strong magnetic coverage on GO	pH—6 Time—50 min Adsorbent—0.05 g Temp—RT	81.21% removal; 2.03 mg/g	(Nguyen et al. 2024)
NiFe_2_O_4_-chitosan-GO	Co (II)	- Strong chelation via -NH_2_/-OH groups - Magnetic separation	pH—9 Time—24 min Adsorbent—0.5 g	96.87% removal; 387.48 mg/g	(Guo & Bulin 2024)
Fe_3_O_4_–CMC-GO	Cu (II)	- High porosity - Fe_3_O_4_ magnetic - CMC adds -COOH	pH—6–7 Temp—45 °C	199.98 mg/g	(Chen et al. 2021)
Poly(vinylimidazole-allylamine)-Fe_3_O_4_-CuS-GO composites	Cd (II) Pb (II)	- Super paramagnetic - Mesoporous	pH—6 Time—80 min Adsorbent—0.4 g Temp—25 °C	Cd (II)—202.24 mg/g Pb (II)—114.05 mg/g	(Moradi et al. 2026)
Fe_3_O_4_@C-GO-PDA	Zn (II) Cu (II) Pb (II) Cd (II)	- PDA adds catechol/amine functional groups - Surface functional groups (–NH₂, C––O, –COOH) of adsorbents	pH—6 Adsorbent—10 mg	99.1% 97.7% 94.6% 91.1% removal efficiencies	(Wang et al. 2025)

##### Crosslinked MGO composites

Crosslinked MGO/GO frameworks introduce mechanically robust, porous network structures that prevent sheet collapse, increase accessible binding sites, stabilize functional groups, and strengthen mechanical moduli, thereby enhancing adsorption capacity and enabling multi-mode interactions such as hydrogen bonding and electrostatic or π–π interactions (Kaur et al. 2025; Tran et al. 2023). A reusable and stable MGO adsorbent was synthesized by using n-propyltrimethoxysilane as a cross-coupling agent. Silica coating of Fe_3_O_4_ protected the magnetic core from corrosion and oxidation.

The prepared magnetic GO was readily isolated from its aqueous solutions using a strong magnet. It also demonstrated excellent adsorption performance for Cd (II) and Pb (II), with the highest possible capacities of 128.2 and 385.1 mg/g, respectively, under neutral circumstances. However, these amidation reagents are prohibitively expensive, preventing magnetic GO from finding practical industrial uses (Bao et al. 2020).

#### Biopolymer composites

To circumvent the drawbacks of (complex separation and agglomeration) GO layers and to improve adsorption performance, GO sheets are incorporated with biopolymers (El-Sheikh et al. 2025). In a recent study, CS-GO-SH composite was synthesized through covalent interaction and electrostatic self-assembly to adsorb Cu (II), Pb (II), and Cd (II) in single- and multi-metal ion environments. A simple diazonium chemical technique was established for generating GO-SH by incorporating SH molecules onto GO sheets. The GO-SH was then employed to self-assemble with CS using electrostatic contact, yielding CS-GO-SH with multifunctional groups (-OH, -COOH, -SH, and -NH_2_). Furthermore, a unique ternary mesoporous composite (GO-CS-PANI) demonstrated outstanding Cr (VI) removal performance both in static and dynamic adsorption, with a maximum absorption of 539.8 mg/g (Rout & Jena 2023).

#### Conducting polymer composites

Graphene oxide (GO) combined with conducting polymers (polyfuran, PANI, PPy, PEDOT, PTh, and their derivatives) forms a class of hybrid adsorbents with excellent capacity for heavy metal removal. GO provides a high surface area and abundant oxygen functional groups that bind metals via complexation and electrostatic attraction. In contrast, conducting polymers provide excellent redox activity, abundant heteroatom functional groups, and tunable electron density (Achary et al. 2021; Joshi et al. 2022; Zhang et al. 2020). For example, the GO-PANI composite attained a maximum adsorption capacity of 1416 mg/g for Pb (II), 2.3 times that of PANI (Xie 2023). Furthermore, the PPy-CS-GO composite electrode exhibited an adsorption capacity of 35.5 mg/g, approximately 2.03 times higher than that of the PPy electrode (17.5 mg/g). In addition, no significant decline in adsorption capacity was observed over multiple adsorption–desorption cycles (Juan’s Qin et al. 2020).

### rGO-based composites

Reduced graphene oxide (rGO)-based composites have emerged as highly effective adsorbents for heavy-metal remediation owing to their enhanced surface area and tunable surface chemistry (Babaei et al. 2025; Uysal et al. 2025). Reduced graphene oxide integrated with polythiophene and silica achieved high removal efficiencies for Pb (II) and Cd (II), reaching 98.7% and 93.2%, respectively, underscoring the role of sulfur-rich polymer matrices in strengthening coordination with soft metal ions (Joshi et al. 2022). Additional studies highlight that functionalizing rGO surfaces significantly improved affinity for heavy metals. For instance, PMMA-rGO-Ag_2_O membrane achieved 99% Cd (II) from aqueous solution with high flux, cost-effectiveness, and facile synthesis technique, making it a promising solution for Cd (II) remediation through filtration and adsorption (Muhammad et al. 2020).

### GQDs-based composites

GQDs-based composites (functionalized GQDs, GQDs–polymer networks, and inorganic composites) offer tunable, high-capacity, and selective adsorption of heavy metals from wastewater (Abdelsalam et al. 2023). A cost-effective GQDs-QS adsorbent demonstrated enhanced removal of Hg (II) and Pb (II) from water, with maximum capacities of 24.7 and 24.9 mg/g, respectively, outperforming untreated quartz sand. The negatively charged GQDs-QS surface at pH > 4 facilitated electrostatic attraction of cationic ions. Reduced efficiency in basic conditions was related to the precipitation of metal hydroxides/oxides, highlighting its potential for industrial wastewater treatment (Mohammad-rezaei & Jaymand 2018).

## Adsorption mechanisms

Adsorption of pollutants into a solid adsorbent involves three basic processes:

- Pollutants are transported to the adsorbent surface through aqueous solution,
- Solid surface adsorption, and
- Internal particle movement (Chai et al. 2021).

Electrostatic interactions, metal ion complexation, or chelation are the primary mechanisms for metal ion adsorption on graphene-based materials. Furthermore, covalent binding, heavy metal coordination, and ion exchange all play important roles in heavy metal adsorption (Ding et al. 2023).

**Fig. 10:**
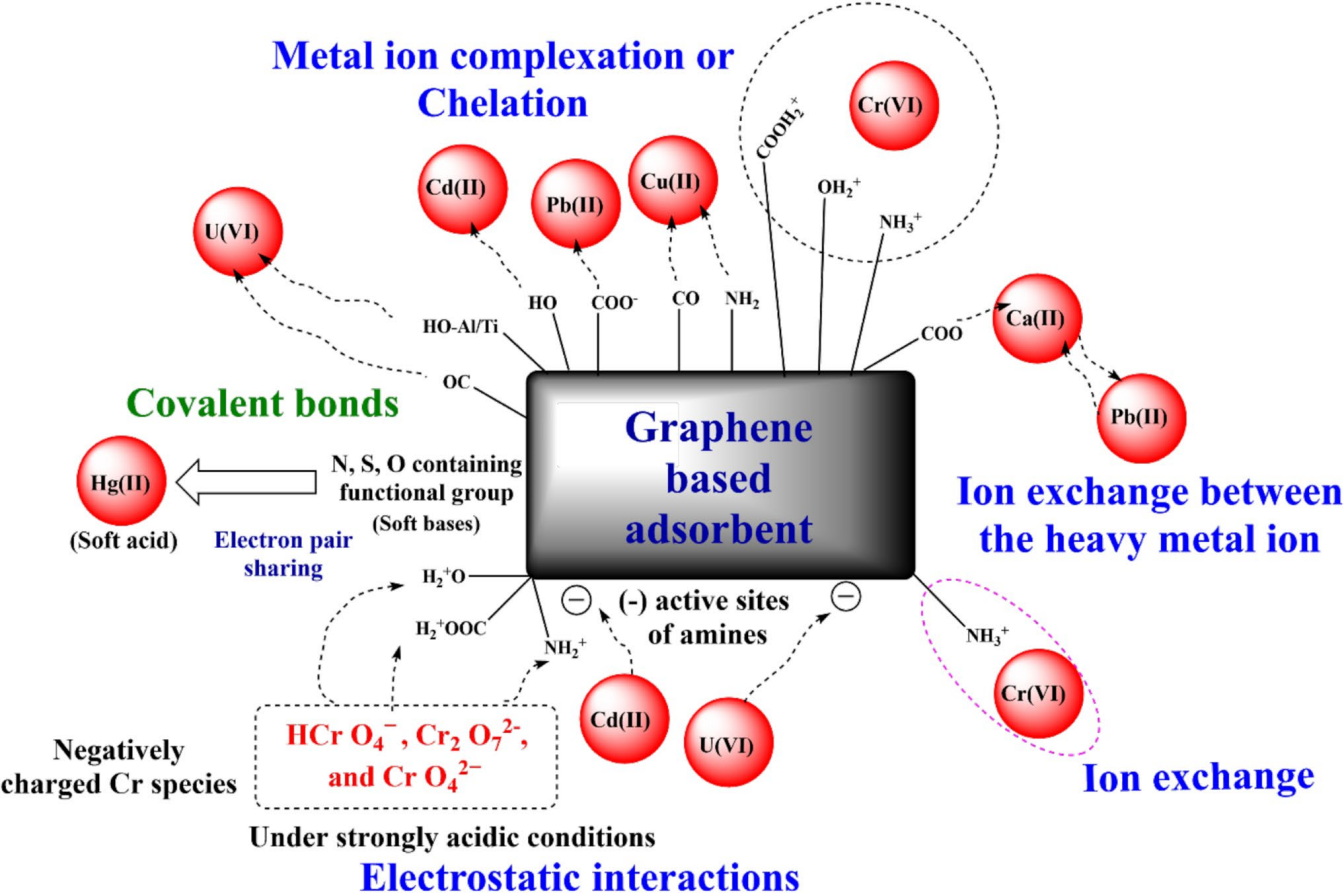
Heavy metal adsorption mechanism of graphene and its derivative composites

Heavy metal adsorption on graphene and its derivative composites is the synergistic effect of several mechanisms. For example, Pb (II) and Cu (II) adsorption onto MWCNT-PDA-GO hybrid aerogels is mainly driven by surface complexation, metal ion coordination, chelation, and electrostatic interaction. Higher pH enhances the electrostatic interaction between heavy metal ions and hybrid aerogels. The aerogels demonstrate greater affinity for Pb (II) than Cu (II), reflecting the metal electronegativity (Zhan et al. 2019). Figure 10 demonstrates the heavy metal adsorption mechanism of graphene and its derivative composites.

### Covalent bonding

Covalent bonding between heavy metals and graphene-based adsorbents involves the chemical interaction of metal ions with functional groups on the modified graphene surface, often through electron donation from oxygen- or nitrogen-containing groups (Ahmad et al. 2020; Liu et al. 2022a, b). For instance, adsorption of Hg (II) on Cyst-prGO is proposed by covalent interaction, and primary heteroatoms (S, N, and O) in the adsorbent play significant roles in the adsorption mechanism. Furthermore, the electronegativity of the heteroatoms contributes to the overall performance. Electron donors follow the electronegativity trend S < N < O, meaning oxygen atoms hold lone pair electrons more tightly than nitrogen or sulfur due to stronger nuclear attraction. Consequently, sulfur donates its lone pair electrons more readily than nitrogen or oxygen, making it more effective in forming interactions with Hg (II) (Yap et al. 2019).

### Chelation or metal ion complexation

**Fig. 11:**
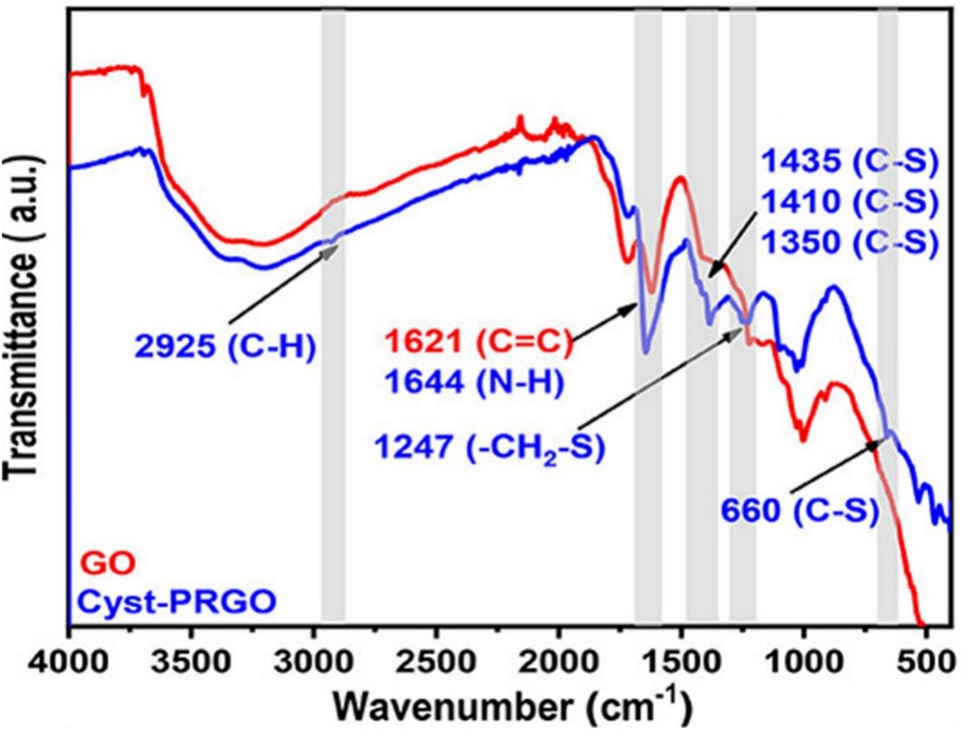
FT-IR spectra of GO and Cyst-prGO. Reproduced with permission from Yap et al. (2019)

Chelating compounds are ligands that provide a more complicated electron donor to the core metal (Al-Salman et al. 2023). The presence of carboxyl, hydroxyl, and N-containing groups in the adsorbent contributes to heavy metal adsorption via chelation (Wu et al. 2019). For example, C–OH groups play a critical role in Cd (II) and Pb (II) adsorption on Fe_3_O_4_-SiO_2_-GO. The most COO − groups onto GO are linked to Fe_3_O_4_-SiO_2_ in the coupling reaction, and the chelating interaction occurs between the metal ions and the carboxyl groups on the adsorbent (Bao et al. 2020). In another study, complexation of Hg (II) with -NH_2_ and -COOH functional groups of Cyst-prGO was evidenced by the FTIR analyses (Fig. 11) (Yap et al. 2019).

### Electrostatic interaction

Electrostatic interaction is a fundamental heavy metal adsorption mechanism for graphene and its derivative composites. This interaction is determined not only by the surface charge and functional group of the adsorbent but also by the pH and ionic strength of the solution (Gupta et al. 2021; Wang et al. 2023; Zaimee et al. 2021). For example, Rout and Jena revealed Cr (VI) adsorption through electrostatic interaction between negatively charged HCrO_4_^−^, Cr_2_O_7_^2−^, and CrO_4_^2−^ ions and protonated hydroxyl, carboxyl, and amine groups of the GO-CS-PANI composite adsorbent under strongly acidic conditions (Rout & Jena 2023). Another research investigated the electrostatic interaction of positively charged Cr (VI) ions and -COO^−^ functional groups of GO-SiO_2_ composites. However, the adsorption is controlled by surface contact and complexation, as the form of Cr (VI) varies at different pHs (Dan et al. 2023). AL-Salman and his group explored the electrostatic interaction between positively charged Cd (II) ions with negatively charged active sites of the amine of CS-graphene nanocomposite, besides the chelation interaction (Al-Salman et al. 2023). Furthermore, electrostatic interaction between negatively charged GO-Ti_x_All_-x_O_y_ particles and positively charged U (VI) was also reported in another research at pH 4 (Ding et al. 2023).

### Ion exchange

Ion exchange is an additional approach to heavy metal adsorption, which involves the exchange of ions between the functional groups present on the graphene-based adsorbent surface and the heavy metal ions in solution. For instance, Cr (VI) adsorption on GO-CS–PANI composite occurs through ion exchange interaction in addition to metal ion complexation, electrostatic, and π–π interaction. The -OH, -NH_2_, and -COOH groups donate electrons to bind Cr (VI), with -NH_2_ groups being especially effective due to the lower electronegativity of nitrogen. Therefore, Cr (VI) replaces amine groups via ion exchange to boost adsorption efficiency (Rout & Jena 2023).

## Factors affecting adsorption

The heavy metal adsorption onto graphene-based materials depends on various factors such as initial pollutant concentration, the amount of adsorbent used, duration of contact, solution pH, temperature, the electronegativity of the heavy metal ions, and the presence of competing ions in the solution.

### Solution pH

The pH of the solution plays a crucial role in heavy metal adsorption, as it affects the surface charge and functional groups of the adsorbent, as well as the ionization state of the metal ions (Chen et al. 2022; Kong et al. 2021; Nizam et al. 2022). At low pH, functional groups of adsorbents are protonated and can easily adsorb negatively charged heavy metal ions (HCrO_4_^−^) via electrostatic interaction. Conversely, as the pH increases, the concentration of -OH ions in the solution increases, and the number of protonated hydrogen ions gradually decreases, resulting in weak adsorbent-pollutant interactions. For example, the highest Cr (VI) adsorption on GO-CS-PANI was reported at pH 2.0 (Rout & Jena 2023). This finding is supported by the literature (Dan et al. 2023; Mahvi et al. 2021).

**Fig. 12:**
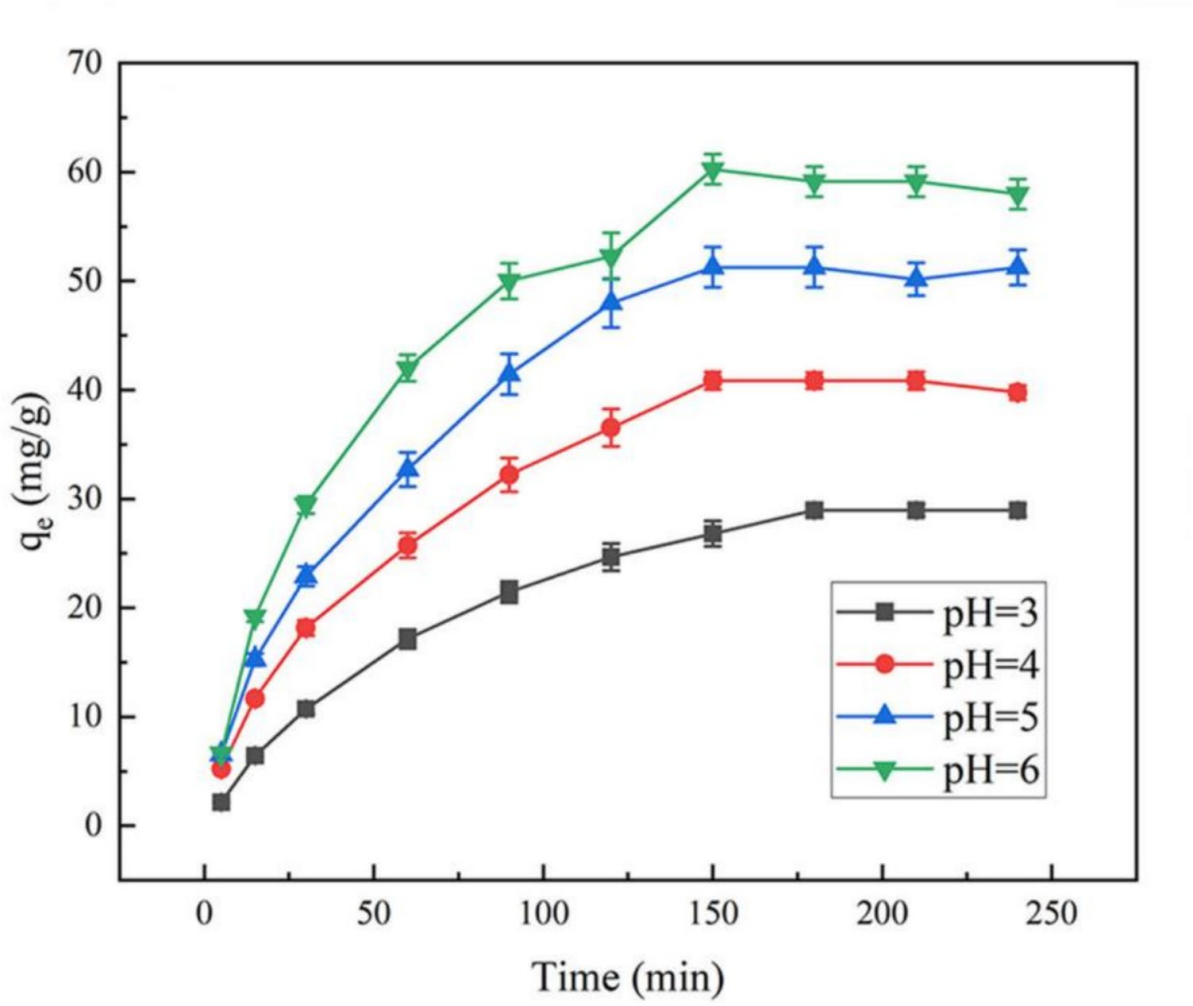
Effect of pH on Cu (II) adsorption on SGCS-GO composite (dosage—1 g/L, temp—30 °C, Cu (II) concentration—20 mg/L). Reproduced with permission from Zhang et al. (2024)

**Fig. 13:**
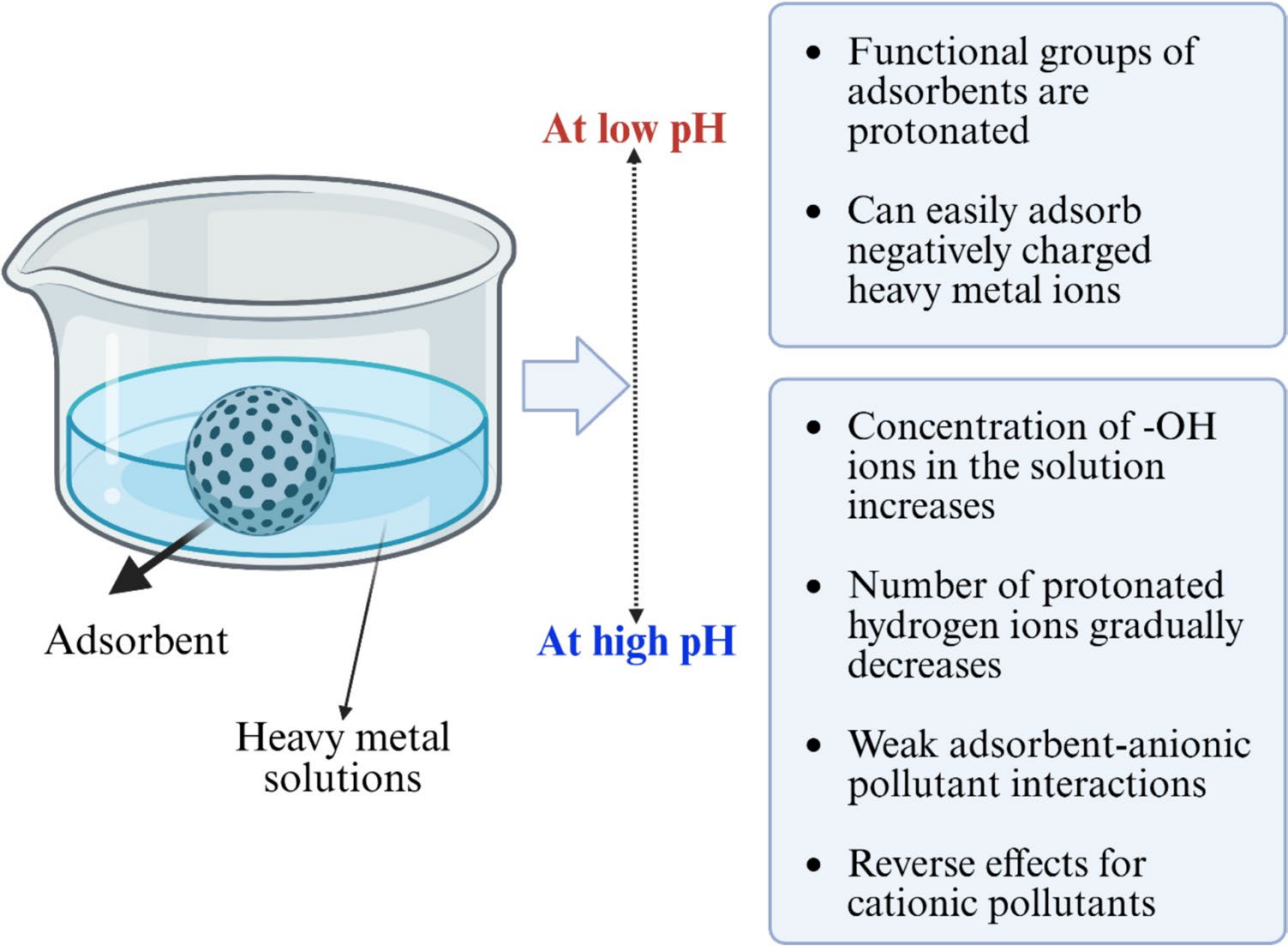
Effect of pH on heavy metal adsorption onto graphene-based adsorbents

However, the effect is reversed for cationic pollutants. For example, increasing the pH significantly improves Pb (II) and Cd (II) adsorption by GO-g-P4VP-PAA hydrogel, as fewer carboxyl groups are protonated and more binding sites become available. Optimal adsorption occurs at pH 5 for Pb (II) and pH 6 for Cd (II); above these values, metal ions precipitate as hydroxides (Zhang et al. 2023). In another study, adsorption of Cu (II) doubled (28.9 to 60.3 mg/g) when pH increased from 3 to 6 in an SGCS-GO composite (Fig. 12) (Zhang et al. 2024). The findings were corroborated by previous studies (Al-Salman et al. 2023; Yap et al. 2019). Figure 13 depicts the effect of pH on heavy metal adsorption on the graphene-based adsorbent.

### Initial concentration of pollutants

**Fig. 14:**
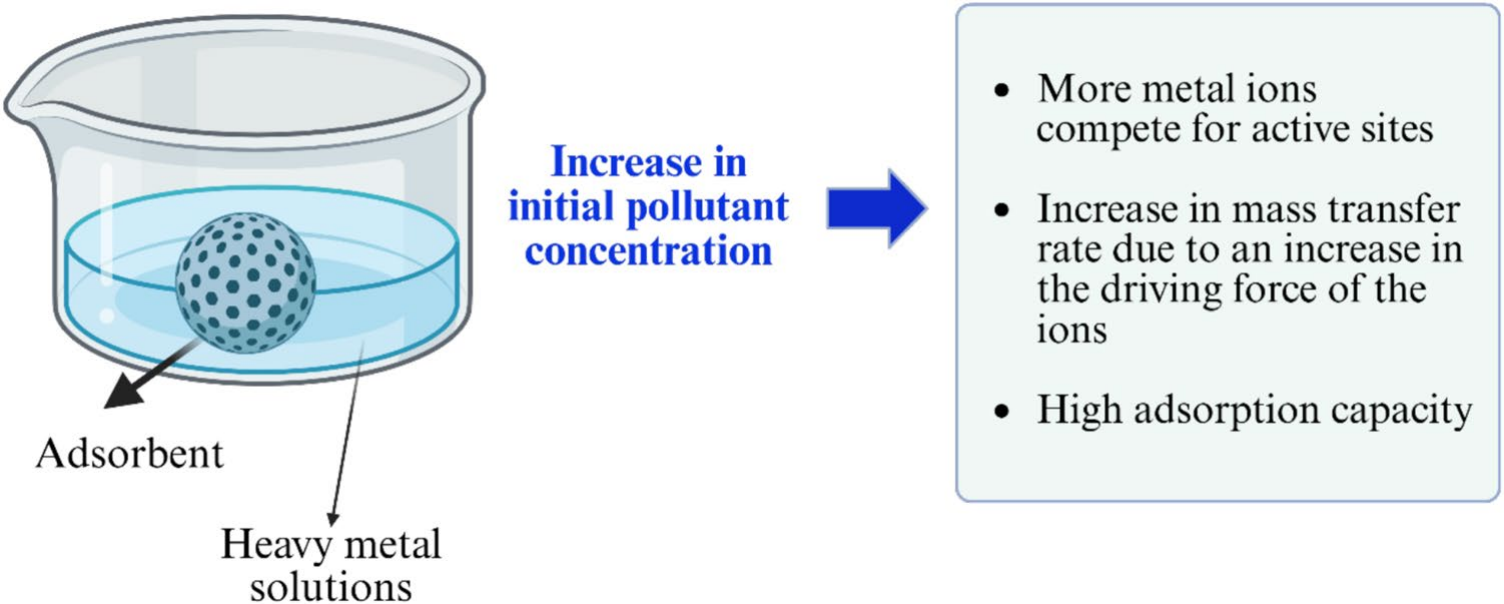
Effect of initial concentration of pollutants on adsorption

As the concentration of metal ions increases at a given adsorbent dosage, the active sites on the adsorbent eventually become saturated. Beyond this point, further increases in metal ion concentration do not enhance the adsorbent’s effectiveness due to inadequate adsorption sites. When the initial concentration of heavy metal ions increases, more ions compete for the available active sites. Additionally, the greater concentration gradient boosts the mass transfer rate, leading to higher adsorption capacity (Fig. 14) (Jiang et al. 2020; Mahmoodi et al. 2020).

For instance, when initial concentrations of Cu (II) and Pb (II) were increased from 50 to 400 mg/L, the adsorption capacities of MWCNT-PDA-GO hybrid aerogel increased 4.3 and 4.7-fold, respectively (Zhan et al. 2019).

### Contact time

Contact time plays a key role in adsorption behavior. Initially, adsorption is rapid due to the abundance of available surface sites and the high concentration of heavy metal ions, which increases interactions with the adsorbent. As adsorption progresses, the remaining sites become harder to access because of repulsive forces and the declining concentration of metal ions, leading to a slower adsorption rate over time (Arshad et al. 2019; Bao et al. 2020).

For example, the SA-PAM-GO composite hydrogel adsorbed Cu (II) and Pb (II) within 60 min, steadily increasing until equilibrium was attained (Jiang et al. 2020). Another study explored 45 min as the best Cr (VI) adsorption time for the GO-CS-PANI composite (Rout & Jena 2023). Furthermore, Cd (II) adsorption on CS-graphene nanocomposite granules was investigated for durations of 30, 60, 120, and 240 min. After 120 min, nearly all active amine sites formed electrostatic interactions with Cd (II), leaving only a small number of available sites and causing the remaining adsorption to proceed more slowly (Al-Salman et al. 2023).

### Ionic strength of solution

Natural water and wastewater contain various electrolyte ions at different concentrations, which can affect how adsorbent surface charges interact with heavy metal ions and, consequently, impact the efficiency of heavy metal adsorption (Velusamy et al. 2021; Zhang et al. 2023).

Recent studies examined how different electrolyte ions affected Pb (II) and Cd (II) adsorption on GO-g-P4VP-PAA hydrogel. Increasing NaCl concentration did not impact on the hydrogel’s ability to adsorb either heavy metal. In contrast, CaCl_2_ had little effect on Pb (II) adsorption but significantly reduced Cd (II) uptake as the CaCl_2_ concentration increased. This difference is linked to the hydration radii of the ions: Pb (II) has the smallest, followed by Ca (II) and Cd (II). A smaller hydration radius made ion exchange between similarly charged ions easier and enhanced adsorption, while a larger radius hindered it. Additionally, ions with smaller hydration shells decreased swelling pressure in the hydrogel and improved its adsorption efficiency (Zhang et al. 2023). Furthermore, the solution’s ionic strength also provides insight into the adsorption mechanisms of heavy metals. Typically, outer-sphere surface complexation and ion exchange are influenced by the change in ionic strength, while inner-sphere surface complexation remains unaffected (Ding et al. 2023; Liu et al. 2022a, b).

### Temperature and adsorption thermodynamics

Temperature significantly affects heavy metal adsorption on graphene-based materials. Higher temperatures enhance adsorption rates by modifying chemical interactions, improving solubility, and either increasing the availability of active sites on adsorbent surfaces or accelerating the diffusion of metal ions (Arshad et al. 2019; Bai et al. 2020).

For instance, Cr (VI) adsorption on the GO-CS-PANI composite increased as the temperature rose to 35 °C and then leveled off at higher temperatures. This improvement was due to greater molecular and ionic movement at elevated temperatures, which boosted the kinetic energy and accelerated the interaction between Cr (VI) ions and the adsorbent surface, enhancing adsorption efficiency (Rout & Jena 2023).

Furthermore, the adsorption capacity of the adsorbent at various temperatures depends on its surface heterogeneity, which leads to differences in the energy levels of its active sites. At lower temperatures, ions are initially captured by sites with lower activation energy. As the temperature rises, sites with higher activation energy become engaged. Additionally, elevated temperatures also help to remove the hydration shells around cations, making them smaller and more mobile, which further enhances physical adsorption (Mahvi et al. 2021).

#### Adsorption thermodynamics

Thermodynamic studies offer detailed information on the intrinsic energy changes that occur during adsorption. The thermodynamic parameters, e.g.,

- Standard enthalpy change (∆*H*, kJ/mol)
- Standard entropy change (∆*S*, J/(mol. K)
- Standard free energy change (Δ*G*, kJ/mol) is evaluated by the following equations:
  $$\mathrm{ln}K= \frac{\Delta S}{R}-\frac{\Delta H}{\mathrm{R}\mathrm{T}}$$
  $$\Delta G= -\mathrm{R}\mathrm{T}\mathrm{l}\mathrm{n} K$$
  $$\Delta G= \Delta H-T\Delta S$$

where *K* is the thermodynamic equilibrium constant, *T* is the temperature in Kelvin (K), and *R* (8.314 Jmol^−1^ K^−1^) is the gas constant. The Δ*H* and Δ*S* are calculated from the slope and intercept of a linear plot of ln *K* versus T^−1^ (Bao et al. 2020).

A positive ∆*H* indicates that adsorption is endothermic, while a positive ∆*S* reflects greater randomness or freedom for the adsorbed molecules. Conversely, a negative entropy value suggests that the process, driven by enthalpy, results in reduced disorder. Enthalpy and Gibbs free energy values also help to identify the nature of adsorption taking place. Negative ∆*G* values signify that adsorption is spontaneous and occurs quickly. Furthermore, if ∆*G* becomes more negative as temperature rises, it means that higher temperatures favor the adsorption process (Mahvi et al. 2021). This occurs because higher temperatures boost the mobility of adsorbate molecules, leading to greater adsorption capacity (Arshad et al. 2019). For instance, Cr (VI) adsorption on the GO-CS-PANI composite is endothermic (∆*H* = 86.18 kJ/mol). The increasing negativity of ∆*G* with temperature indicates improved adsorption temperatures. Furthermore, elevated temperatures enhance the movement of Cr (VI) ions to the adsorbent surface, boosting their interaction and overall adsorption efficiency (Rout & Jena 2023). Table 7 summarizes the thermodynamic parameters of heavy metal adsorption on graphene and its derivatives composites.

**Table 7:** Thermodynamic parameters of heavy metal adsorption on graphene and its derivative composites

Adsorbent	Adsorbate	Temperature (K)	$\Delta G$(kJ mol^−1^)	$\Delta H$(kJ mol^−1^)	$\Delta S$ (kJ K^−1^ mol^−1^)	Ref
Fe_3_O_4_-GO	Cr (VI)	288	− 3.16	55.18	0.201	(Mahvi et al. 2021)
		298	− 5.69			
		308	− 8.11			
		318	− 9.27			
Fe_3_O_4_-SiO_2_-GO	Cd (II)	298	− 0.92	16.49	0.300	(Bao et al. 2020)
		308	− 1.36			
		318	− 2.10			
Fe_3_O_4_-SiO_2_-GO	Pb (II)	298	− 7.94	81.70	0.060	(Bao et al. 2020)
		308	− 11.14			
		318	− 13.96			
GO embedded calcium alginate beads	Pb (II)	303	− 24.41	− 16.80	0.025	(Arshad et al. 2019)
		313	− 24.63			
		323	− 24.55			
		333	− 25.28			
GO embedded calcium alginate beads	Hg (II)	303	− 23.99	− 10.72	0.044	(Arshad et al. 2019)
		313	− 24.43			
		323	− 24.87			
		333	− 25.31			
GO embedded calcium alginate beads	Cd (II)	303	− 24.31	− 49.35	0.064	(Arshad et al. 2019)
		313	− 24.95			
		323	− 25.59			
		333	− 26.23			
rGO-oCNT-PANI nanocomposite	Pb (II)	298	− 22.3	36.90	0.159	(Ali et al. 2022)
		308	− 23.9			
		318	− 26.3			
rGO-oCNT-PANI nanocomposite	Zn (II)	298	− 4.97	26.60	0.150	(Ali et al. 2022)
		308	− 5.28			
		318	− 5.52			

### Adsorbent dosages

The dosage of adsorbent is a critical factor affecting heavy metal adsorption efficiency. Determining the optimal amount ensures effective interaction between metal ions and available adsorption sites. While higher adsorbent dosages enhance removal by providing more binding sites, they also reduce adsorption capacity per unit mass (Liu et al. 2020). For example, the removal efficiencies of Cu (II) and Pb (II) increased with increasing dosage of the GO-based adsorbent (GO-TETA-DAC) from 10 to 60 mg (Yao et al. 2019). Table 8 demonstrates the effect of adsorbent dosages on heavy metal adsorption.

**Table 8:** Impact of varying adsorbent amounts on adsorption efficiency

Adsorbent	Adsorbate	Adsorbent dosages (g/L)	Efficiency or removal (%)	Adsorption capacity (mg/g)	Ref
Fe_3_O_4_-GO	Cr (VI)	0.10	49.6	496.0	(Mahvi et al. 2021)
		0.60	82.1	83.3	
GO-CS–PANI composite	Cr (VI)	0.20	75.2	265.0	(Rout & Jena 2023)
		0.40	96.5	190.0	
GO-g-P4VP-PAA hydrogel	Pb (II)	0.50	60.0	425.0	(Zhang et al. 2023)
		2.50	97.5	150.0	
	Cd (II)	0.25	50.0	400.0	
		3.00	95.0	75.0	
GEINs-BC	Pb (II)	0.05	51.6	-	(Yan & Li 2022)
		0.25	96.7		

### Graphene content in adsorbent

The adsorption of heavy metals by graphene-based composites is strongly influenced by the graphene content. Higher graphene content typically enhances adsorption capacity due to increased surface area and active sites, but excessive content can lead to aggregation and reduced performance (Al-Salman et al. 2023). For instance, increasing GO loading in a ZIF-L/GO composite from 20 to 50 resulted in higher adsorption capacities for Pb (II) (from 172.4 to 188.7 mg/g), compared to the base ZIF-L material (81.4 mg/g) (Ahmad et al. 2021).

### Coexisting matters

Contaminated water contains diverse ions and compounds that compete for adsorption sites (Jiang et al. 2020; Wu et al. 2019). For instance, Cyst-prGO demonstrates a clear hierarchy in metal affinity: Hg (II) > Pb (II) > Cu (II) > Cd (II). In tests with equal concentrations of Hg (II) and three competing ions, removal efficiencies were 83% for Hg (II), 15% for Pb (II), 12% for Cu (II), and 0% for Cd (II), highlighting its strong preference for Hg (II) (Yap et al. 2019). Therefore, competitive adsorption studies are necessary to understand how coexisting substances influence metal ion removal. A recent experiment investigated how magnetic CMC-SA-GO-Fe_3_O_4_ gel adsorbs Cu (II), Cd (II), and Pb (II) when common ions like Na^+^, K^+^, Mg^2+^, and Ca^2+^ (10 mmol/L) are present. The results showed that these competing ions had little effect on Pb (II) adsorption, but they significantly reduced the adsorption of Cu (II) and Cd (II) (Wu et al. 2019).

**Fig. 15:**
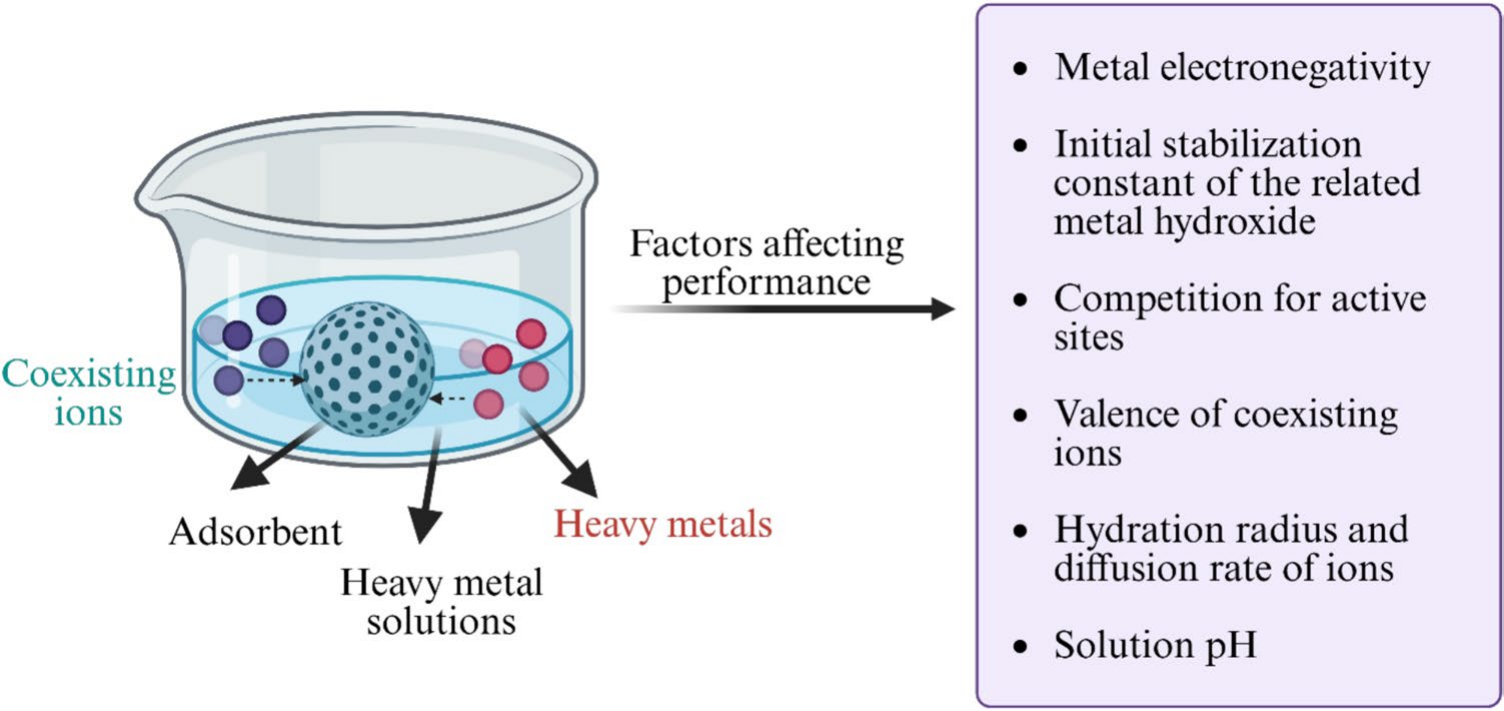
Effect of coexisting ions on heavy metal adsorption on graphene-based adsorbent

For Cr (VI) adsorption using GO-CS-PANI composite, HCO_3_^−^ and SO_4_^2−^ anions significantly hindered adsorption, while Cl^−^ and NO_3_^−^ had little effect; Co^2+^ and Cu^2+^ cations also showed no notable impact (Rout & Jena 2023). For Fe_3_O_4_-GO, adsorption decreased in the presence of NaCl, NaNO_3_, and Na_2_CO_3_ due to competition for active sites, with Cl^−^ ions having a stronger effect because of their faster diffusion (Mahvi et al. 2021). In uranium adsorption by GO-Ti_x_Al1_-x_O_y_, most cations (K^+^, Na^+^, Mg^2+^, Ca^2+^, and Zn^2+^) had little effect, but Ca^2+^ reduced adsorption due to competition and its large hydration radius. Anions like CO_3_^2−^ and PO_4_^3−^ also lowered uranium uptake by forming stable complexes, yet overall uranium adsorption remained above 80%, demonstrating the composite’s strong potential for selective heavy metal removal (Ding et al. 2023; Li et al. 2022a, b; Wang et al. 2021a, b; Yin et al. 2021). Figure 15 demonstrates the effect of coexisting ions on heavy metal adsorption on a graphene-based adsorbent.

## Desorption and regeneration of the adsorbent

A major environmental issue with adsorbents is the generation of large amounts of sludge, which can be addressed by recycling the adsorbent through multiple cycles. From a commercial standpoint, the key factors for any modern adsorbent are its desorption and regeneration capabilities (Mahvi et al. 2021; Rout & Jena 2023).

Regenerating the adsorbent involves removing heavy metals (desorption) with an eluent solution. For uranium desorption, pure water, H_2_O_2_, Na_2_CO_3_, and H_2_O_2_/Na_2_CO_3_ mixture achieve elution efficiencies of 1.0%, 43.1%, 69.3%, and 96.7%, respectively. The high elution efficiency of H_2_O_2_/Na_2_CO_3_ solution is related to the formation of uranium-peroxy carbonate. GO-Ti_x_Al_1-x_O_y_ maintains over 90% uranium adsorption after five cycles and shows no leaching of Ti^4+^ or Al^3+^, indicating strong regeneration and durability (Ding et al. 2023). However, magnetic adsorbents are easier to regenerate than other types, as they can be quickly separated using an external magnetic field and offer excellent stability and reusability (Bao et al. 2020). Table 9 demonstrates the regeneration performance of graphene and its derivatives composite.

**Table 9:** Regeneration performance of graphene-based composites

Adsorbent	Adsorbate	Eluents for the desorption process	No. of cycles	Removal efficiency (%)	Ref
GO-CS-PANI composite	Cr (VI)	0.1 M NaOH	5	Reduces from 96.5 to 76.2	(Rout & Jena 2023)
GO-Ti_x_Al_1-x_O_y_	U (VI)	Pure water, 0.1 M H_2_O_2_, 1.0 M Na_2_CO_3_, mixed solution of H_2_O_2_ (0.1 M) and Na_2_CO_3_ (1 M)	5	> 90	(Ding et al. 2023)
GO-g-P4VP-PAA hydrogel	Pb (II) Cd (II)	0.5 M HCl	5	> 80	(Zhang et al. 2023)
Fe_3_O_4_-GO	Cr (VI)	**-**	6	> 90	(Mahvi et al. 2021)
SA-PAM-GO hydrogel	Cu (II) Pb (II)	1 M HCl	5	Cu (II) = > 80 and Pb (II) = 60	(Jiang et al. 2020)
Magnetic Fe_3_O_4_-SiO_2_-GO composites	Cd (II) Pb (II)	0.8 M HCl	12	95	(Bao et al. 2020)
GOCA bead	Pb (II) Hg (II) Cd (II)	HCl (pH 5) and CaCl_2_ (200 ppm) mixture	5	75–80	(Arshad et al. 2019)
Cyst-prGO	Hg (II)	Thiourea in 0.1 M HCl eluent	5	> 97	(Yap et al. 2019)
Thiol and amido modified alginate-based GO hydrogel	Pb (II) Cu (II)	0.1 M HCl	5	> 90	(Zhang et al. 2022)
PDA @ Fe₃O₄ @ C-GO	Zn (II) Cu (II) Pb (II) Cd (II)	Ethyl acetate	5	> 95	(Wang et al. 2025)

## Conclusions

Graphene and its derivatives’ composites have shown greater potential as adsorbents for heavy metals. The primary mechanisms responsible for heavy metal adsorption on graphene-based materials include electrostatic interaction, surface complexation, ion exchange, and covalent interaction. However, the effectiveness of adsorption is influenced by several factors. These include the physicochemical characteristics of the adsorbent and various operational parameters. Key operational factors affecting adsorption capacity are the pH of the solution, adsorption time, the initial concentrations of metal ions, the adsorbent amount, the ionic strength of the solution, the proportion of graphene in the composite, temperature, and the presence of other ions in the solution. All these aspects play a significant role in determining overall adsorption efficiency. Despite previous efforts, certain significant issues remain unresolved.

**Fig. 16:**
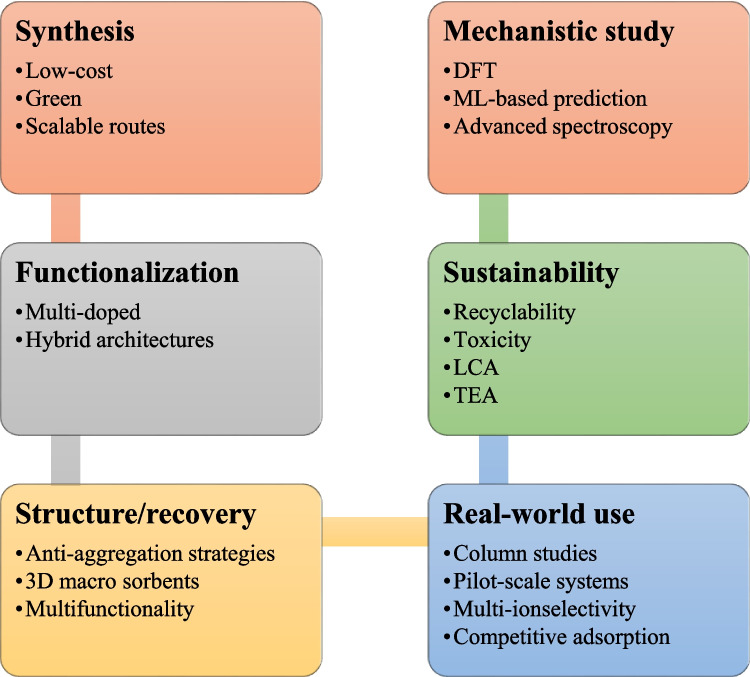
Future research focuses on graphene-based composites for heavy metal adsorption

- Most research was conducted on a lab scale. The use of graphene and its derivative composites in pilot-scale or real-world wastewater scenarios have received little attention.
- Researchers continue to face challenges in producing graphene-based adsorbents on a large scale at a reasonable cost.
- To effectively remove numerous contaminants from industrial effluent, studying multicomponent solutions is important.
- Despite research into adsorbent regeneration and reuse, nothing has been determined about the future of utilized adsorbents.
- Conducting a life cycle assessment and techno-economic study on developed adsorbents can help determine their suitability for various applications.
- Further research is needed to assess the toxic levels of graphene-based composites to the ecosystems. If these gaps are filled, graphene and its composites will dominate water filtration technology soon. Figure 16 summarizes the future research directions of graphene and its derivative-based composites for heavy metal adsorption.

## Data Availability

Available from the corresponding author on reasonable request.

## Abbreviations

AC: Activated carbon
BC: Biochar
CNTs: Carbon nanotubes
CVD: Chemical vapor deposition
CS: Chitosan
CS-GO-SH: CS-sulfydryl-functionalized graphene oxide
Cyst-prGO: Cysteamine modified partially reduced graphene oxide
CMC: Carboxymethyl cellulose
CMC-SA-GO-Fe_3_O_4_: Carboxymethyl chitosan–sodium alginate–graphene oxide-Fe_3_O_4_
DFT: Density functional theory
DTPA: Diethylenetriaminepentaacetic acid
DAC: Dialdehyde cellulose
FTIR: Fourier transform infrared spectroscopy
GO: Graphene oxide
GQDs: Graphene quantum dots
GO-g-P4VP-PAA: Poly (4–vinylpyridine) grafted graphene oxide–polyacrylic acid
GO-SH: Sulfydryl-functionalized graphene oxide
GO-CMC-PVA: Graphene oxide–carboxymethyl–chitosan-polyvinyl alcohol
GQDs-QS: Graphene quantum dot-coated quartz sand
GOCA: Graphene oxide embedded calcium alginate
GEINs-BC: Graphene encapsulated iron nanoparticles embedded in biochar
LDH: Layered double hydroxides
MD: Molecular dynamics
MOF: Metal-organic framework
MGO: Magnetic graphene oxide
MWCNT-PDA: Polydopamine modified multiwalled carbon nanotubes
PAA: Polyacrylic acid
PDA: Polydopamine
P4VP: Poly (4-vinylpyridine)
PEI: Polyethylenimine
PEDOT: Poly (3, 4-ethylenedioxythiophene)
PG-GO-PEI: Phosphogypsum–graphene oxide–polyethyleneimine
PANI: Polyaniline
PPy: Polypyrrole
PTh: Polythiophene
PAM: Polyacrylamide
rGO: Reduced graphene oxide
SA: Sodium alginate
SA-PAM-GO: Sodium alginate–polyacrylamide–graphene oxide
SNPs: Sulfur nanoparticles
SF: Silk fibroin
SGCS-GO: Sulfhydryl graphene oxide-chitosan-graphene oxide
TETA: Triethylenetetramine
UV: Ultraviolet
UCN-GH: Ultrathin g-C_3_N_4_–graphene oxide hydrogel
ZIF-L/GO: Zeolitic imidazolate framework-L–graphene oxide
2D: Two-dimensional
3D: Three-dimensional
ML: Machine learning
LCA: Life cycle analysis
TEA: Technoeconomic analysis
